# Reduced *Akr1b7* signaling drives ovarian aging and reproductive dysfunction

**DOI:** 10.1016/j.isci.2026.114719

**Published:** 2026-01-19

**Authors:** Keishiro Isayama, Kenji Watanabe, Masato Ohtsuka, Seisuke Kimura, Tomoaki Murata, Takeshi Honda, Masataka Asagiri, Shun Sato, Hiroshi Tamura, Norihiro Sugino, Yoichi Mizukami

**Affiliations:** 1 Institute of Gene Research, Yamaguchi University Science Research Center, Yamaguchi 755-8505, Japan; 2 Institute of Life Science and Medicine, Yamaguchi University Science Research Center, Yamaguchi 755-8505, Japan; 3 Department of Molecular Life Science, Division of Basic Medical Science and Molecular Medicine, Tokai University School of Medicine, Isehara, Kanagawa 259-1193, Japan; 4 Department of Industrial Life Sciences, Faculty of Life Sciences, Kyoto Sangyo University, Kita-ku, Kyoto 603-8555, Japan; 5 Japan and Center for Plant Sciences, Kyoto Sangyo University, Kamigamo-motoyama, Kita-ku, Kyoto 603-8555, Japan; 6 Department of Pharmacology, Yamaguchi University Graduate School of Medicine, Yamaguchi 755-8505, Japan; 7 Department of Obstetrics and Gynecology, Yamaguchi University Graduate School of Medicine, Yamaguchi 755-8505, Japan

**Keywords:** Health sciences, Endocrinology, Endocrine system physiology, Female reproductive endocrinology, Endocrine regulation

## Abstract

Natural ovarian aging is associated with a progressive decline in female fertility. Here, we comprehensively analyzed RNA expression during ovarian aging in mice during the estrous cycle following ovulation stimulation. The transient activation of the Aldo-keto reductase *Akr1b7* pathway observed in the ovaries of young mice was absent in older mice. *Akr1b7*^−/−^ mice exhibit attenuated oocyte Akt activation, impaired follicular development, an increased proportion of ovulated immature oocytes, and decreased litter size. The estrous cycle is extended in *Akr1b7*^−/−^ mice due to a prolonged diestrous stage, driven by sustained progesterone levels. This elevation in progesterone was associated with the reduced expression of *Cyp17a1*, a progesterone-metabolizing enzyme in the *Akr1b7*-positive theca cell layers. Together, these findings identify *Akr1b7* as a regulator of ovarian signaling, hormone homeostasis, and reproductive function, with the disruption of this pathway producing phenotypes associated with declining fertility.

## Highlights

• *Akr1b7* loss impairs Akt activation in primordial follicles and disrupts oocyte maturation
• *Akr1b7* deficiency accelerates immature oocyte ovulation and reduces litter size
• *Akr1b7* deletion decreases *Cyp17a1* expression and prolongs diestrus by elevating progesterone
• Reduced *Akr1b7* signaling recapitulates ovarian aging phenotypes in mice

## Graphical abstract

**Figure:**
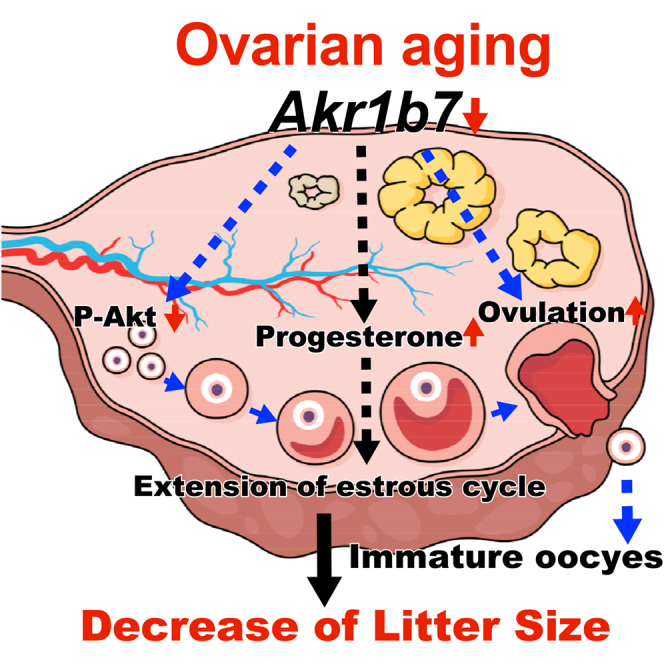

## Introduction

The ovary is a female-specific reproductive organ that serves as the primary source of oocyte supply.^1^ The oocyte is released from the mature follicle, which develops from primordial follicles within the ovary in association with the surrounding somatic cells.^2^^,^^3^ During folliculogenesis, primordial follicles are activated and progress sequentially through primary and secondary stages, accompanied by granulosa cell proliferation and the recruitment of steroidogenic theca cells.^1^ The formation of antral follicles, characterized by a fluid-filled cavity adjacent to the oocyte, precedes ovulation. Following ovulation, residual follicular cells differentiate into corpora lutea (CLs), which produce estrogen and progesterone.^4^ Progesterone levels are maintained following fertilization but decline rapidly due to the regression of CLs in the absence of fertilization.^5^ Primordial follicles are generated before birth and not during an individual’s lifetime. Specifically, 1–2 million oocytes are present in the ovary at birth, decreasing to 300,000–400,000 primordial follicles by puberty, and gradually declining throughout the reproductive age.^6^^,^^7^ Although ovulation ceases at menopause, residual oocytes often remain in the ovary, suggesting that age-related changes in the follicular microenvironment and somatic cell function contribute to reproductive senescence. Clinically, ovarian aging is associated with attenuated ovulatory and steroidogenic responses to human chorionic gonadotropin (hCG) stimulation.^8^^,^^9^ Mice and rats have been established as models for studying ovarian aging in humans.^3^ Signs of ovarian aging, such as a decreased number of developing follicles and a prolonged estrous cycle, were observed in mice aged approximately 28–48 weeks. Subsequently, cessation of the estrous cycle was observed at 44–72 weeks^3^^,^^10^ Notably, aged mice retain residual oocytes despite diminished ovulatory capacity. Inhibiting apoptosis in *Bax*-deficient mice prolongs the ovarian lifespan,^11^ highlighting the importance of follicle pool preservation in delaying reproductive aging. Ovarian function is regulated by coordinated signaling between oocytes and somatic cells, mediated by gonadotropins and steroid hormones. Transcriptomic analyses have revealed age-associated changes in oocytes, granulosa cells, and theca cells.^12^^,^^13^^,^^14^ Oxidative stress in oocytes and granulosa cells is closely associated with ovarian aging, impaired growth, and disrupted redox homeostasis.^14^ In theca and interstitial cells of the ovary, LH induces the expression of *aldo-keto reductase (Akr)1b7*, also known as mouse vas deferens protein, MVDP,^15^ which is closely associated with serum progesterone levels.^16^ In humans, AKR1B1, the functional ortholog of mouse *Akr1b7*,^17^ is downregulated in senescent proximal tubule epithelial cells.^18^ However, the role of *Akr1b7* signaling in coordinating follicular development and age-associated reproductive decline at the whole-ovary level remains unclear. However, the signal transduction pathways and activated molecules influencing ovarian aging during folliculogenesis remain unclear in the whole ovary, where cell-to-cell interactions are essential.

In this study, we comprehensively analyzed mRNA expression in mouse ovaries according to the time course during folliculogenesis to elucidate the signaling pathway affecting ovarian aging. Sustained suppression of *Akr1b7* was observed in the ovaries of aged mice, in contrast to its transient expression in young mice. Using *Akr1b7*^−/−^ mice, generated by deleting the start codon through *improved* genome editing via the oviductal nucleic acid delivery (i-GONAD) method, we demonstrate that loss of *Akr1b7* disrupts ovarian signaling, resulting in impaired follicular development, ovulation of immature oocytes, altered estrous cyclicity, and reduced fertility.

## Results

### Ovulation and follicular development in the aged ovaries

**Figure 1:**
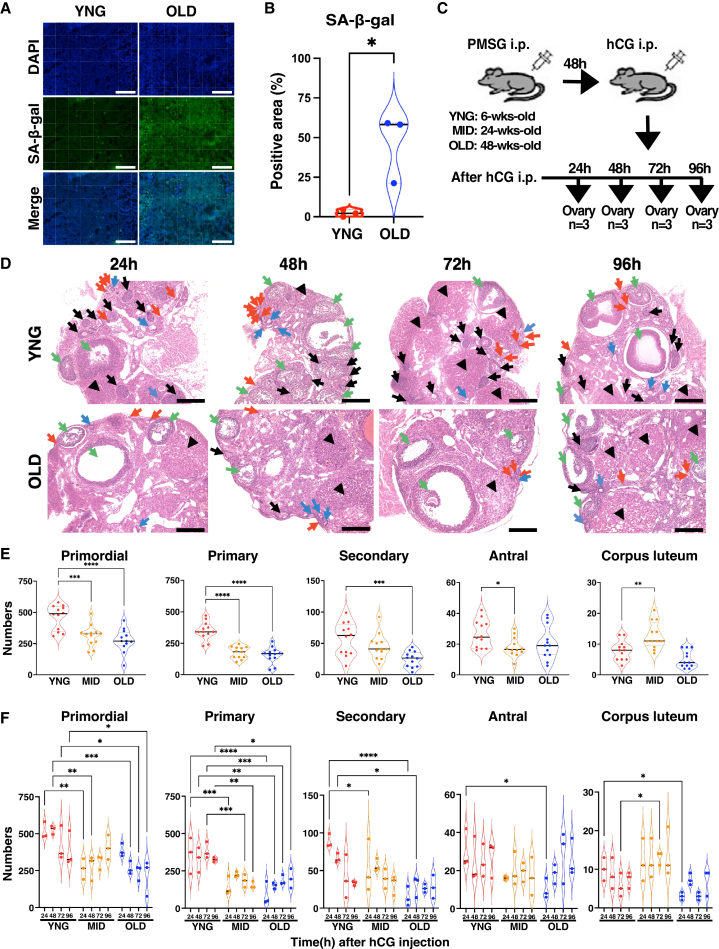
Follicular development in ovarian aging after ovulation stimulation (A) Representative SA-β-gal staining of ovarian sections collected from 6-week-old and 48-week-old female mice at diestrus. Scale bars, 200 μm. (B) Violin plots show the SA-β-gal-positive area (%) relative to the total area. *n* = 3, ∗*p* < 0.05; two-tailed Student’s *t* test. (C) Sampling design for ovaries after ovulation stimulation. YNG, MID, and OLD female mice were injected intraperitoneally with 5 IU-PMSG and 5 IU-hCG; ovaries were collected at the indicated time (*n* = 3). Of the two ovaries, one was used for histological analysis and the other for RNA expression analyses. (D) Representative H&E staining of ovarian sections from YNG and OLD mice after PMSG/hCG injection. Follicles are indicated with arrows as follows: primordial (red), primary (blue), secondary (black), and antral (green). Corpus lutea are indicated with black arrowheads. Scale bars, 200 μm. (E) Violin plots show the total numbers of each follicle type and corpora luteum in serial sections from YNG, MID, and OLD mice. *n* = 3. ∗*p* < 0.05, ∗∗*p* < 0.01, ∗∗∗*p* < 0.001, and ∗∗∗∗*p* < 0.0001; one-way ANOVA followed by the Dunnett’s multiple test. (F) Violin plots show the numbers of each follicle stage and CLs in serial sections in YNG, MID, and OLD mice after the PMSG/hCG injection. *n* = 3, ∗*p* < 0.05, ∗∗*p* < 0.01, ∗∗∗*p* < 0.001, and ∗∗∗∗*p* < 0.0001; two-way ANOVA followed by the Tukey’s multiple comparisons test.

The number of fetuses in female mice, along with a senescence marker, was used to determine the age in weeks of mice exhibiting ovarian aging. Six-to eight-week-old female mice carried an average of approximately 8 fetuses. Whereas fetuses were rarely observed in mice older than 48 weeks. SA (senescence-associated)-β-gal, a marker of senescent cells, was observed in the stromal cells of 48-week-old mice; however, the staining was rarely detected in 6-week-old mice (Figures 1A and 1B). Similarly, 8-OHdG (8-hydroxy-2′-deoxyguanosine), an oxidative stress (DNA damage) marker, was detected in the stromal cells surrounding the follicles of 48- to 56-week-old mice, but not in 6-week-old mice (Figures S1A and S1B). Follicular development during the estrous cycle was examined after pregnant mare serum gonadotropin (PMSG) and hCG treatment in young (6- to 8-week-old), middle-aged (24-week-old), and old mice (48- to 56-week-old) (Figure 1C). The ovaries of the young mice contained more follicles than those of the middle-aged and old mice, except for antral follicles (Figures 1D, 1E, and S1C). Follicular counts during the diestrous stage in mice with natural ovulation (Figure S1D) were similar to those observed in mice with superovulation (Figure 1E). To evaluate the follicular response to ovulation induction, we quantified follicles at different developmental stages following PMSG/hCG injection (Figure 1F). Since primordial follicles cannot mature within 96 h, this analysis reflects the dynamics of follicles already in the growth phase. In young mice, follicle numbers decreased over time, consistent with ovulation and luteinization. In contrast, aged mice exhibited minimal reduction in early-stage follicles, indicating impaired follicular progression. Specifically, although primordial follicles continued to decline until 48 h in aged mice, the number of more advanced follicles increased compared to the 24-h time plot, suggesting abnormal or delayed development (Figure 1F). To confirm the ovarian reserve, ovulated oocytes were examined in mice following ovarian induction. In aged mice, the number of ovulated oocytes was significantly reduced, and the incidence of immature ovulation was increased, although a small number of oocytes remained. (Figures S1E and S1F). These findings suggest that follicular developmental dysfunction in aged ovaries may arise during the primordial follicle developmental stage.

### Whole-transcriptome analysis during the estrous cycle in ovarian aging

**Figure 2:**
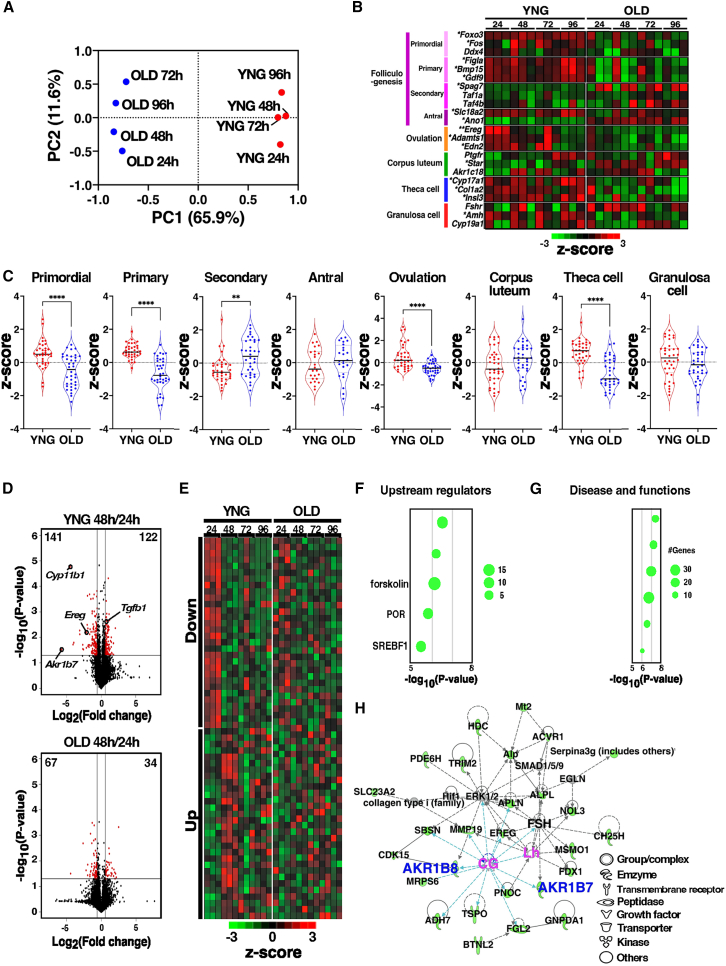
Whole transcriptome analysis of ovarian aging after ovulation stimulation (A) Principal component analysis of mRNA expression values in the ovaries of YNG and OLD. *n* = 3, plotted using the values calculated from PC1 and PC2. (B) CPM values of the indicated marker genes were transformed to log_2_ and normalized by *Z* score, as shown in the heatmap. (C) Z-scored values of the marker genes indicated in (B) were plotted for each group. ∗∗*p* < 0.01, ∗∗∗∗*p* < 0.0001; two-tailed Student's *t* test. (D) Volcano plot shows differentially expressed genes in ovaries 24−48 h after PMSG/hCG injection. The negative log_10_-transformed *p*-values are plotted against the log_2_-transformed fold changes. Genes with a fold change of >1.5 and a *p*-value below 0.05 are plotted in red circles. (E) Heat maps show the z-scores of the genes indicated by the red circles of (D). (F) The gene set identified as downregulated in (D) was analyzed using IPA software, and the detected upstream regulators are shown. (G) The downregulated gene set from (D) was analyzed using IPA, and pathways related to diseases and functions are shown. (H) The gene set from (D) was analyzed using IPA software, and a representative pathway is shown.

To elucidate the signaling pathways involved in follicular development dysfunction in aged mice, whole-transcriptome analysis was performed on mouse ovaries during the estrous cycle following ovulation treatment. Over 15,000 genes were detected from approximately 30 million reads in each ovary, and the expression patterns of representative genes closely matched those obtained by quantitative polymerase chain reaction (PCR; Figure S2). Principal component analysis (PCA) revealed that ovarian gene expression patterns in young and old mice were separated by the first principal component (PC1), regardless of the estrous cycle. In young mice, gene expression following ovulation stimulation shifted in a positive direction along the second principal component (PC2), whereas in old mice, the 96 h samples shifted back toward the negative direction on PC2 (Figure 2A). Marker genes associated with stages of follicular development, identified using the single-cell dataset^14^^,^^19^^,^^20^ demonstrated that *Ere*g, *Adamts*1, and *Edn2,* the genes involved in ovulation, exhibited transiently high expression 24 h after stimulation in young mice, whereas their expression remained nearly constant throughout the cycle in old mice (Figures 2B and 2C). Marker genes for primordial follicles (*Foxo3*, *Fos*, and *Ddx4*), primary follicles (*Figla*, *Bmp15*, and *Gdf9*), and theca cells (*Cyp17a1*, *Col1a2*, and *Insl3*) were consistently expressed at high levels in the ovaries of young mice; however, in old mice, their expression during the estrous cycle was sustained but at low levels (Figures 2B and 2C). CG-responsive genes *Cyp11b1* and *Ereg* showed a rapid decrease 48 h after stimulation, whereas *Tgfb1* was immediately induced by stimulation (Figure 2D). In old mice, the responsive genes were only mildly regulated or showed no response to CG (Figure 2B). Alterations in gene expression during the estrous cycle were evident in young mice at 48 h after stimulation, whereas these changes were markedly weaker in old mice (Figures 2D and 2E). The 141 genes transiently upregulated 24 h after stimulation were analyzed using Ingenuity Pathway Analysis (IPA), which identified CG as the primary upstream regulator (Figure 2F). The downstream effectors indicated that steroid hormone pathways play a central role in follicular development in response to CG (Figure 2G). Network analysis illustrated that ovulation stimulation with CG and LH is closely associated with the Aldo-keto reductases AKR1B7 and AKR1B8 (Figure 2H). The expression of *Akr1b7* transiently increased 24 h after ovulation stimulation and rapidly decreased by 48 h, whereas *Akr1b8* expression remained constant throughout the estrous cycle (Figure S3). CG and follicle-stimulating hormone (FSH) reappeared in the network analysis at 96 h poststimulation, indicating entry into the next estrous cycle (Figures S4A–S4C). In old mice, the CG-activated NADPH oxidase pathway was detected in the downregulated genes, and CG and FSH were not observed until 96 h, except following exogenous administration, contrasting with the pathway detected in young mice (Figures S4D–S4F). These observations suggest that decreased *Akr1b7* expression may contribute to follicular developmental dysfunction during ovarian aging.

### *Akr1b7* mRNA expression in ovarian aging

**Figure 3:**
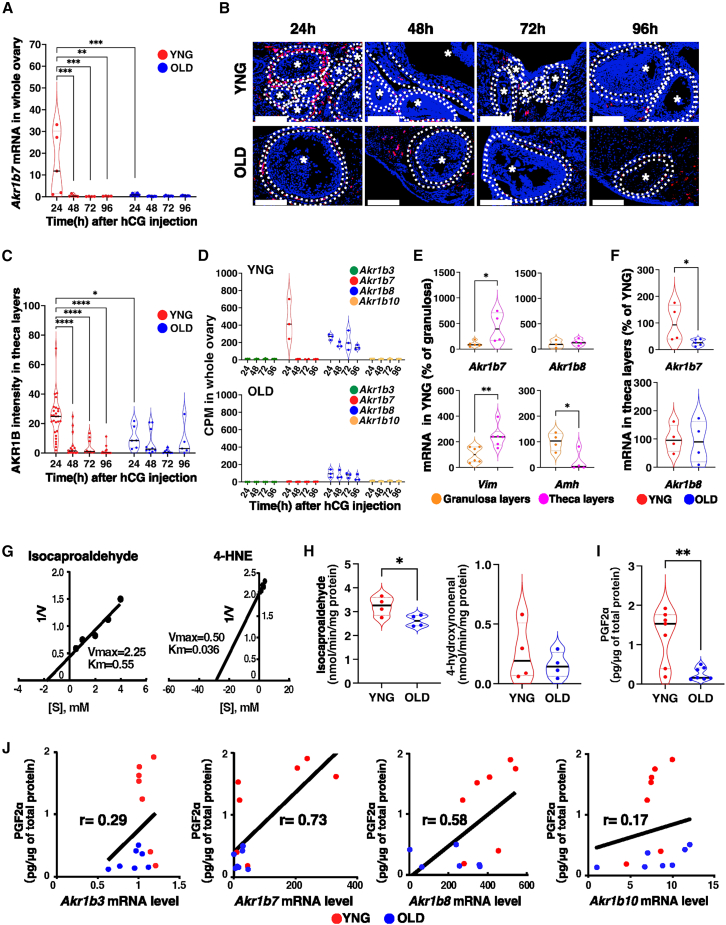
Expression and enzymatic analysis of AKR1B7 in ovarian aging (A) Quantitative PCR analysis of *Akr1b7* mRNA expression was performed using ovaries collected from YNG and OLD female mice at the indicated times after PMSG/hCG injection. Violin plots show the expression level relative to *Gapdh*. n = 4–7, ∗∗*p* < 0.01, ∗∗∗*p* < 0.001; two-way ANOVA followed by the Tukey’s multiple comparisons test. (B) Images show immunohistochemical staining of ovaries from YNG and OLD females after PMSG/hCG injection, using an antihuman AKR1B10 antibody that recognizes an epitope identical to AKR1B7. Sections were incubated with the antibody and visualized using a DAB reaction. Images of the stained section were pseudo-colored with DAB stains (red) and hematoxylin (blue) counterstains. The circle with dotted lines indicates the theca layer. *n* = 3. Scale bars (white line), 100 μm. (C) Mean staining intensities in the theca cell layer from (B) were calculated by dividing the signal intensity by the area. The number of follicles analyzed was 5–26. *n* = 3, ∗*p* < 0.05, ∗∗∗∗*p* < 0.0001; two-way ANOVA followed by the Tukey’s multiple comparisons test. (D) Violin plots show the expression of AKR1B superfamily genes as determined by whole transcriptome analysis. *n* = 3. (E) Violin plots show *Akr1b7* and *Akr1b8* mRNA expression relative to *Actb* in laser micro-dissected theca and granulosa cell layers from YNG and OLD female mice. *Vim* and *Amh* mRNA were measured as marker genes for theca and granulosa cells, respectively. n = 3–6, ∗*p* < 0.05, ∗∗*p* < 0.01; two-tailed Student’s *t* test. (F) Violin plots show *Akr1b7* and *Akr1b8* mRNA expressions relative to *Actb* in theca layers of YNG and OLD mice. ∗*p* < 0.05; two-tailed Student’s *t* test. (G) Lineweaver-Burk plots were generated from the reductase activities for ICA (left panel) or 4-HNE (right panel) in YNG ovaries at 24 h after PMSG/hCG injection. (H) Violin plots show reductase activities for ICA (left panel) or 4-HNE (right panel) in cytosolic extracts of YNG and OLD ovaries 24 h after PMSG/hCG injection. *n* = 4, ∗*p* < 0.05; two-tailed Student’s *t* test. (I) PGF_2α_ concentrations were quantified by ELISA using the ovary homogenates of YNG and OLD mice at 24 h after PMSG/hCG injection. *n* = 7, ∗∗*p* < 0.01; two-tailed Student’s *t* test. (J) mRNA expression levels of the indicated *Akr1b* superfamily were plotted against PGF_2α_ concentration in YNG (red) and OLD (blue) mice. *n* = 7; r indicates the Pearson’s correlation coefficient.

The expression of *Akr1b7* mRNA during follicular development was confirmed by quantitative PCR. High expression was detected 24 h after hCG administration and rapidly declined over the following 24 h, consistent with whole transcriptome analysis (Figure 3A). *Akr1b7* expression in aged mice remained low after stimulation and persisted at low levels throughout the estrous cycle. Immunohistochemical staining using an antibody against human AKR1B10, whose epitope sequence is identical to that of mouse AKR1B7, was performed on the theca cell layer and stromal cells following ovulation stimulation (Figure 3B). Staining in theca cells expressing luteinizing hormone (LH) and CG receptors decreased significantly 48 h after stimulation (Figure 3C), mirroring mRNA expression patterns. To confirm staining detected in the theca cells, we examined gene expression in target regions extracted from ovarian tissue slices via laser microdissection using superfamily specific primers. Among the *Akr1b* superfamily members, *Akr1b7* and *Akr1b8* mRNAs were exclusively expressed in the ovaries of young mice (Figures 3D and S3). *Akr1b7* was primarily expressed in the theca cell layer of the ovaries of young mice, consistent with the expression of *Vimentin (Vim)*, a theca cell marker, and contrasted with the expression of *anti-Müllerian hormone (Amh)*, a granulosa cell marker. *Akr1b8* expression was observed in both the granulosa and theca cell layers (Figure 3E). *Akr1b7* mRNA levels in theca cell layers of old mice were significantly lower than those in the young mice (Figure 3F). Staining with an antihuman AKR1B10 antibody corresponded with *Akr1b7* mRNA expression, indicating that the antibody recognizes mouse AKR1B7 in the ovary. Isocaproaldehyde (ICA) and 4-hydroxynonenal (4-HNE), known substrates for AKR1B7, were used to examine the enzyme’s characteristics in the ovaries. The apparent Km values for ICA and 4-HNE in the ovaries of young mice were 550 μM and 36 μM, respectively (Figure 3G), closely matching the Km values obtained using recombinant AKR1B7, which were 320 μM for ICA and 62 μM for 4-HNE.^21^ In the ovaries of the old mice, the reduction activities of ICA and 4-HNE were slightly lower than those in young mice, and the extent of reduction differed from the levels of *Akr1b7* mRNA observed in the old mice (Figure 3H). Prostaglandin (PG) F_2α_-forming activity was measured in the ovaries after hCG treatment because AKR1B7 catalyzes the conversion of PGH_2_ to PGF_2α_, whereas AKR1B8 shows no activity toward this substrate. PGF_2α_-forming activity in the ovaries of old mice was significantly lower than that of young mice (Figure 3I), and PGF_2α_ production positively correlated with *Akr1b7* mRNA levels with a correlation coefficient of 0.73 (Figure 3J). These findings indicate that the decrease in *Akr1b7* mRNA during ovarian aging is at least partially associated with reduced PGF_2α_-forming activity.

### Generation of *Akr1b7*-deficient mice using oviductal nucleic acid delivery

We generated *Akr1b7* locus-disrupted mice (*Akr1b7*^−/−^) using i-GONAD, a genome editing technique involving the electroporation of preimplantation embryos within the oviduct, to elucidate the physiological roles of AKR1B7 in ovarian aging (Figure S5A). Guide RNA (gRNA) was designed for the target region containing the protospacer adjacent motif sequence located five bases downstream of the *Akr1b7* start codon, and was delivered into the oviduct along with the CAS9 protein (Figure S5B). Fifty-two fetuses were born from six female mice treated with i-GONAD. In 13.5% of the fetuses, both alleles of *Akr1b7* were disrupted near the start codon. Twenty fetuses (23.1%) had a hetero-deletion mutation within the *Akr1b7* locus, and 13 fetuses (25.0%) exhibited mosaicism (Figure S5C). The representative genome sequence of mice with disrupted *Akr1b7* showed a 20-base deletion, including the start codon of *Akr1b7* (Figure S5D). In the ovaries of young wild-type (WT) mice, 24 h after hCG stimulation, reads from whole-transcriptome analysis mapped uniformly across all exons of *Akr1b7*. In *Akr1b7*^−/−^ mice, mapped reads were absent around the start codon of *Akr1b7* exon 1 (Figure S5E), although reads were detected throughout all other regions. AKR1B7 staining, which was primarily detected in the theca cell layer of WT mice, was absent in *Akr1b7*^−/−^ mice (Figures S5H and S5I). The *Akr1b7*^−/−^ mice showed no effects on the expression of other *Akr1b* superfamily members (Figures S5F and S5G). These observations confirm that *Akr1b7*-disrupted mice were successfully generated using the i-GONAD method.

### Ovulation of immature oocytes in *Akr1b7*^−/−^ mice

**Figure 4:**
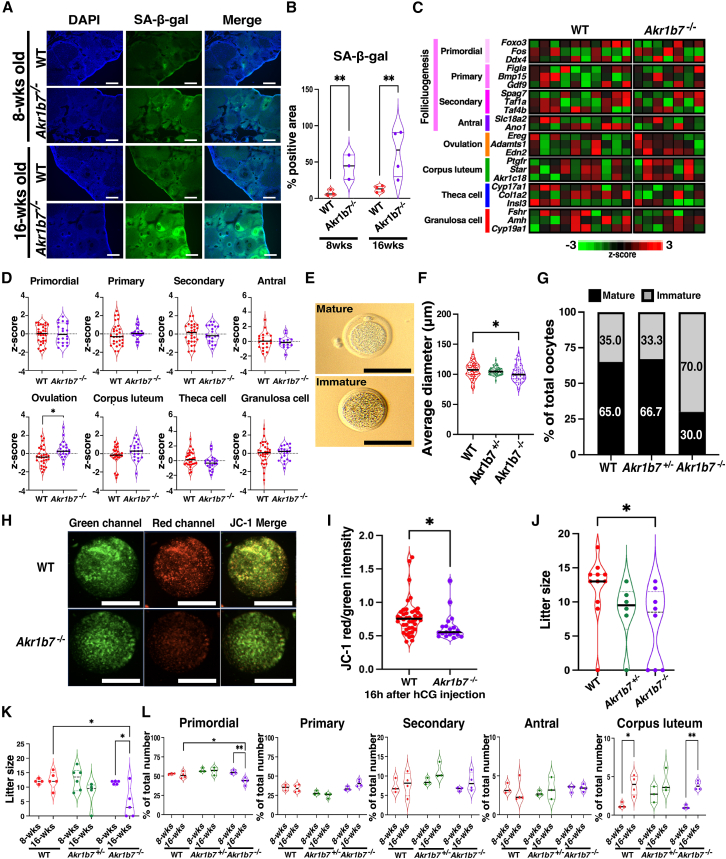
Follicular development in *Akr1b7*-deficient mice after ovulation stimulation (A) Representative SA-β-gal staining of ovarian sections from 8- and 16-week-old WT and *Akr1b7*^−/−^ mice at the diestrous stage. Scale bars, 100 μm. (B) Violin plots show the β-gal-positive area (%) relative to the total area of an ovarian section. n = 3–4, ∗∗*p* < 0.01; two-way ANOVA followed by the Tukey’s multiple comparisons test. (C) Heatmap shows z-scored values of the indicated marker genes in ovaries from YNG WT (*n* = 10) and *Akr1b7*^−/−^ mice (KO) (*n* = 7) at 24 h after PMSG/hCG injection. (D) Violin plots show z-scores of the marker gene set from (C). ∗*p* < 0.05; two-tailed Student's *t* test. (E) Representative images of oocytes obtained from YNG WT (upper) and *Akr1b7*^−/−^ mice (lower) at 16 h after PMSG/hCG injection. Scale bars, 100 μm. (F) Violin plots show the average diameters of oocytes, including the zona pellucida. WT; *n* = 79, *Akr1b7*^+/−^; *n* = 69, *Akr1b7*^−/−^; *n* = 72. ∗*p* < 0.05; one-way ANOVA followed by Dunnett's multiple test. (G) Bar graphs show the rates of mature and immature oocytes relative to total oocytes. WT; *n* = 90, *Akr1b7*^+/−^; *n* = 69, *Akr1b7*^−/−^; *n* = 70. (H) Mitochondrial staining with JC-1 in ovulated oocytes obtained from YNG WT and *Akr1b7*^−/−^ mice at 16 h after PMSG/hCG injection. Green: JC-1 monomer at lower mitochondrial potential, Red: JC-1 aggregates at higher mitochondrial potential. Scale bars, 50 μm. (I) Violin plots show the ratio of JC-1 red to green intensity. WT; *n* = 42, *Akr1b7*^−/−^; *n* = 19, ∗*p* < 0.05; two-tailed Student’s *t* test. (J) Violin plots show the litter size in YNG WT, *Akr1b7*^*+/−*^, and *Akr1b7*^−/−^ mice treated with PMSG/hCG injection. WT; *n* = 11, *Akr1b7*^+/−^; *n* = 6, *Akr1b7*^−/−^; *n* = 8, ∗*p* < 0.05; one-way ANOVA followed by the Dunnett’s multiple comparisons test. (K) Violin plots show litter size from natural mating in 8-week-old and 16-week-old mice of WT, *Akr1b7*^*+/−*^, and *Akr1b7*^−/−^. *n* = 4 or 6 in 8-week-old, *n* = 4 or 5 in 16-week-old, ∗*p* < 0.05; two-way ANOVA followed by the Tukey’s multiple comparisons test. (L) Violin plots show the rates of each follicle type and corpus lutea, counted in serial ovarian sections from 8-week-old and 16-week-old WT, *Akr1b7*^*+/−*^, and *Akr1b7*^−/−^ mice at 24 h after PMSG/hCG injection; *n* = 3 in 8-week-old mice, *n* = 3 or 4 in 16-week-old mice. ∗*p* < 0.05, ∗∗*p* < 0.01; two-way ANOVA followed by the Tukey’s multiple comparisons test.

We examined the relationship between *Akr1b7* and aging during the interphase phase of the ovary to exclude the influence of estrous cycling and ovulation. To assess cellular senescence in ovarian tissue, SA-β-gal assays were performed on the ovaries of WT and *Akr1b7*^−/−^ mice. We found that β-gal activity was significantly higher in *Akr1b7*^−/−^ ovaries at 8 weeks and increased further after 16 weeks (Figures 4A and 4B). These results suggest that senescence-related pathways contribute to the reproductive decline observed in *Akr1b7*^−/−^ mice. The oxidative stress (DNA damage) marker 8-OHdG was also significantly elevated in the ovaries of young *Akr1b7*^−/−^ mice compared with young WT mice (Figures S6B and S6C). Mice from both groups showed no significant differences in the morphology of developing follicles, as observed by H&E staining (Figure S6A). DNA repair- and oxidative stress-associated genes, including *Sod1* and *Il6*, were significantly decreased in the ovaries of young *Akr1b7*^−/−^ mice (Figures S6D–S6F). The reduced gene expression levels resembled those observed in old WT mice, suggesting that *Akr1b7* regulates ovarian aging. To examine the involvement of *Akr1b7* in ovarian aging, we comprehensively analyzed mRNA expression in the ovaries of 6-week-old *Akr1b7*^−/−^ mice. Marker genes related to follicular development, classified using scRNA-seq analysis, showed significant expression of ovulation-associated genes in *Akr1b7*^−/−^ mice (Figures 4C and 4D). Furthermore, the oocytes ovulated in *Akr1b7*^−/−^ mice were smaller than those in the WT mice and were immature, remaining at the germinal vesicle or metaphase I stage without the first polar body, similar to those observed in aged mice (Figures 4E, 4F, and S6H). Immature oocytes comprised approximately 70% of ovulated oocytes from *Akr1b7*^−/−^ mice, compared to approximately 35% in *Akr1b7*^*+/−*^ and WT mice (Figures 4G and S6G–S6I). The average total number of ovulated oocytes per mouse was similar between the *Akr1b7*^*+/−*^, WT, and *Akr1b7*^−/−^ groups (Figure S6I). Oocytes from *Akr1b7*^−/−^ mice showed decreased membrane potential in the mitochondria (Figures 4H and 4I). Consistent with the increase in immature oocytes, litter size was significantly reduced in superovulation-treated *Akr1b7*^−/−^ mice compared to WT mice (Figure 4J). The litter size of *Akr1b7*^*+/−*^ was also reduced, but the difference was not statistically significant among the mice (Figure 4J). In natural mating without superovulation treatment, the litter size was significantly decreased in 16-week-old *Akr1b7*^−/−^ mice (Figure 4K). Analysis of follicular stages in old mice showed a significant decrease in primordial follicles in 16-week-old *Akr1b7*^*+/−*^ mice, whereas no such decrease was observed in 16-week-old *Akr1b7*^*+/−*^ mice or WT mice (Figures 4L and S6J).

### Downstream molecules of AKR1B7 in follicular development

**Figure 5:**
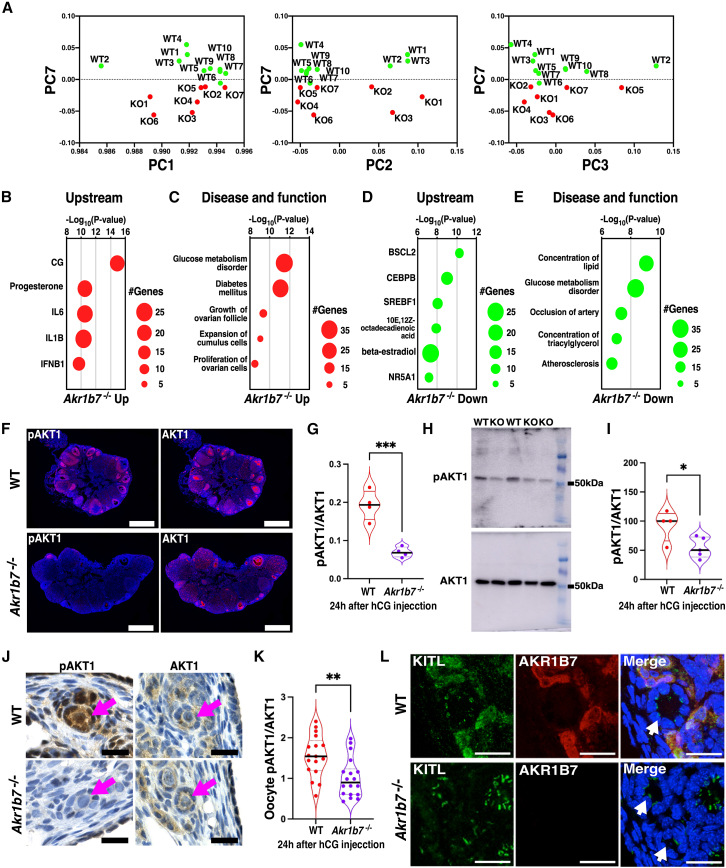
Signal transduction in the primordial follicles of *Akr1b7*-deficient mice after ovulation stimulation (A) Principal component analysis was performed using mRNA expression from ovaries collected from YNG WT (*n* = 10) and *Akr1b7*^−/−^ mice (KO) (*n* = 7) at 24 h after PMSG/hCG injection. Dot plots show the values of PC1 (left panel), PC2 (center panel), or PC3 (right panel) against the values of PC7. (B–E) The upregulated genes (B and C) and downregulated genes (D and E), determined from factor loadings in PC7, were analyzed using IPA software. Dot plots show upstream regulators (B and D) or diseases and functions (C and E). (F–L) Ovaries were collected from YNG WT and *Akr1b7*^−/−^ mice 24 h after PMSG/hCG injection. (F) Immunofluorescence images show AKT1 and pAKT1 staining. Scale bars, 500 μm. (G) Violin plots show the ratio of pAKT1/AKT1 in (F) *n* = 4, ∗∗∗*p* < 0.001; two-tailed Student’s *t* test. (H) Ovaries were subjected to immunoblotting using anti-AKT1 or anti-pAKT1 antibodies (n = 4–5). (I) Violin plots show the ratio of band intensities of pAKT1 and AKT1 in (H). ∗*p* < 0.05; two-tailed Student’s *t* test. (J) Representative DAB staining images of AKT1 and pAKT1 in ovaries. The red arrow indicates a primordial follicle. Scale bars, 20 μm. (K) Violin plots show the ratio of pAKT1 to AKT1 intensities in oocytes of primordial follicles in Figure S7A *n* = 17–18, ∗∗*p* < 0.01; two-tailed Student’s *t* test. (L) Immunofluorescence images show staining with Nuclei-DAPI (blue), KITL-TRITC (green), and AKR1B7-Cy5 (red). The white arrow indicates a primordial follicle. Scale bars, 40 μm. *n* = 4.

To elucidate the signaling pathway underlying AKR1B7-activated folliculogenesis, we performed PCA on RNA-seq data from the ovaries of *Akr1b7*^−/−^ mice. The PCA plots revealed a distinct difference in gene expression between WT and *Akr1b7*^−/−^ mice along PC7 (Figure 5A), and the loading factors driving this separation were used for IPA pathway analysis. The activated genes in *Akr1b7*^−/−^ mice indicated that CG acts as an upstream molecule, suggesting enhanced ovulation stimulation in *Akr1b7*^−/−^ mice (Figure 5B). Analysis of effector pathways in *Akr1b7*^−/−^ mice revealed the upregulation of glucose metabolism disorders and diabetes mellitus (Figure 5C). Analysis of the downregulated genes indicated the inhibition of glucose and lipid metabolism, along with a decrease in steroid-associated transcription factors (Figures 5D and 5E). The pathways detected in *Akr1b7*^−/−^ mice closely resembled those detected in the ovaries of the old mice (Figures 2F and 2G). Akt was significantly activated in the whole ovary in response to hCG; however, this activation was absent in *Akr1b7*^−/−^ mice (Figures 5F–5I). Within the ovary, Akt activation occurred in the oocytes of primordial follicles and in the granulosa cell layers of developing follicles in WT mice. However, this activation was attenuated in *Akr1b7*^−/−^ mice (Figures 5J, 5K, S7A, and S7B). In primordial follicles, extracellular signaling molecules are required to activate Akt by *Akr1b7*, which is selectively expressed in stromal cells, and the tissue distribution of the Akt-activating c-Kit ligand (KITL) was examined.^22^ KITL staining was observed in the cytoplasm of *Akr1b7*-expressed stromal cells, as well as in the punctate pattern of granulosa cells, and only the KITL signal in *Akr1b7*-expressing cells was lost in *Akr1b7*^−/−^ mice (Figure 5L). At 16 weeks of age, KITL staining in granulosa cells was attenuated in *Akr1b7*^−/−^ mice, consistent with a decrease in the number of primordial follicles (Figure S7C). *Akr1b7* regulates the Akt activation required for follicle development through KITL in stromal cells.

### Extension of estrous cycles in *Akr1b7*^−/−^ mice

**Figure 6:**
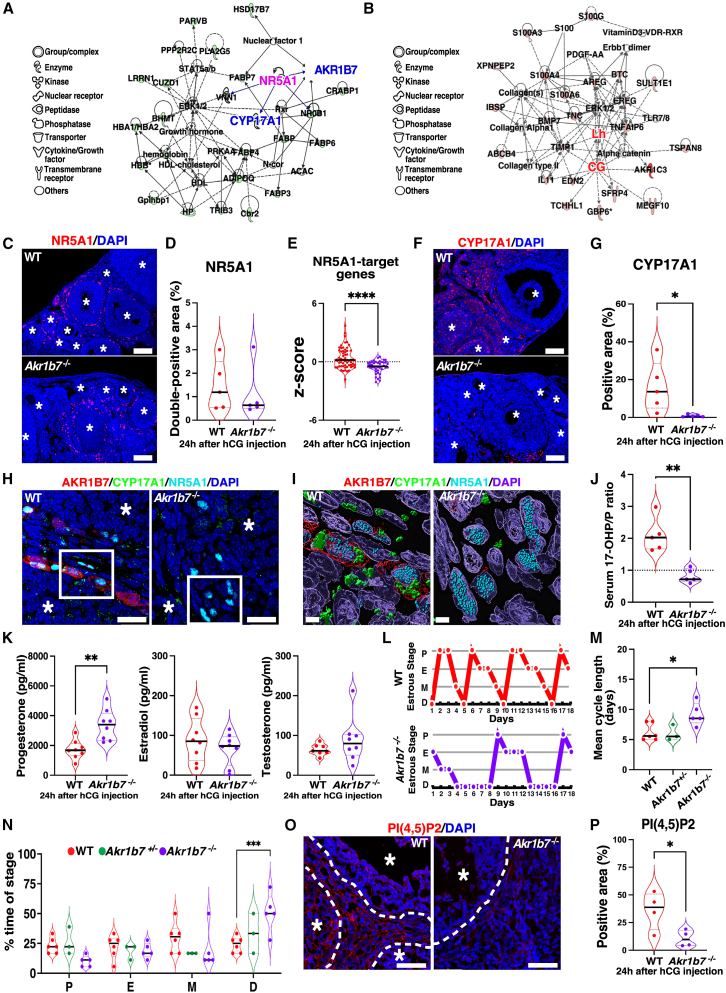
Signaling pathway in thecal cell layers in *Akr1b7*-deficient mice after ovulation stimulation (A and B) Networks show pathways analyzed using downregulated genes (A) and upregulated genes (B) determined from factor loadings in PC7. (C) Immunofluorescence images show NR5A1 staining (red) in YNG WT and *Akr1b7*^−/−^ ovaries 24 h after PMSG/hCG injection. Asterisks indicate growing follicles at the secondary or antral stage. Scale bars, 100 μm. Nuclei-DAPI (blue). *n* = 5. (D) Violin plot shows the nuclear NR5A1-positive area (%). (E) Violin plots show the z-scores from RNA-seq analysis of NR5A1 downstream genes (*Cyp17a1, Cyp21a1*, *Cyp26b1*, *Lhcgr*, *Nr0b1*, *Vnn1*). WT; *n* = 10, KO; *n* = 7. ∗∗∗∗*p* < 0.0001; two-tailed Student's *t* test. (F) Immunofluorescence images show CYP17A1 staining (red) in YNG WT and *Akr1b7*^−/−^ ovaries at 24 h after PMSG/hCG injection. Asterisks indicate grows follicles at the secondary or antral stage. Scale bars, 100 μm. Nuclei-DAPI (blue). *n* = 5. (G) Violin plot shows CYP17A1-positive area (%). ∗*p* < 0.05; two-tailed Student's *t* test. (H) Confocal images show nuclei-DAPI (blue), CYP17A1-FITC (green), NR5A1-TRITC (cyan), and AKR1B7-Cy5 (red). Asterisks indicate growing follicles at the secondary or antral stage. Scale bars, 20 μm. The white squared area indicates the theca cell layers. (I) Immunofluorescence images processed into 3D using Imaris software, showing nuclei-DAPI (blue), CYP17A1-FITC (green), NR5A1-TRITC (cyan), and AKR1B7-Cy5 (red). Scale bars, 4 μm. (J) Violin plots show the 17-OHP/progesterone ratio in the serum of YNG WT and *Akr1b7*^−/−^ mice 24 h after PMSG/hCG injection. *n* = 5, ∗∗*p* < 0.01; two-tailed Student’s *t* test. (K) Violin plots show serum progesterone, estradiol, and testosterone concentrations, quantified by ELISA in YNG WT and *Akr1b7*^−/−^ mice 24 h after PMSG/hCG injection. *n* = 8, ∗∗*p* < 0.01; two-tailed Student’s *t* test. (L) Representative estrous cycle patterns, determined by vaginal cytology, are shown for YNG WT and *Akr1b7*^−/−^ mice. Proestrus; P, estrus; E, metestrus; M and diestrus; D, n *=* 5–6. (M) Mean estrous cycle length in YNG WT, *Akr1b7*^*+/−*^, and *Akr1b7*^−/−^ mice. ∗*p* < 0.05; one-way ANOVA followed by the Dunnett’s multiple comparisons test. (N) Violin plots show the percentages of time spent in each stage of the estrous cycle in YNG WT, *Akr1b7*^*+/−*^, and *Akr1b7*^−/−^ mice. ∗∗∗*p* < 0.001; one-way ANOVA followed by the Dunnett’s multiple comparisons test. (O and P) Immunofluorescence images show PIP2 staining (red) in YNG WT and *Akr1b7*^−/−^ ovaries 24 h after PMSG/hCG injection. Asterisks indicate grows follicles at the secondary or antral stage. The dotted lines indicate the boundary between theca and granulosa layers. Scale bars, 50 μm. Nuclei-DAPI (blue) (P) Violin plots show the PIP2-positive area (%) in the total area of an ovarian section in Figure 7O *n =* 4. ∗*p* < 0.05; two-tailed Student’s *t* test.

Network analysis in *Akr1b7*^−/−^ mice suggested that NR5A1 directly suppressed *Cyp17a1* expression, influencing progesterone metabolism within the network that includes *Akr1b7* (Figure 6A); LH and CG stimulation enhanced the expression of ovulation-associated genes, such as *Ereg* and *Areg* (Figure 6B). Immunohistochemical analysis demonstrated that NR5A1 was present in theca cell layer surrounding developing follicles, with similar staining detected in *Akr1b7*^−/−^ mice (Figures 6C and 6D). The expression of NR5A1-target genes was significantly decreased in the ovaries of *Akr1b7*^−/−^ mice (Figure 6E), suggesting AKR1B7 may regulate the activation of NR5A1 without altering its expression. Staining for CYP17A1, an NR5A1-target gene, was absent in theca cell layers of the ovary in *Akr1b7*^−/−^ mice, in contrast to WT mice (Figures 6F, 6G, and S7D). CYP17A1 colocalized with AKR1B and NR5A1 in theca cell layers of developing follicles (Figures 6H and 6I). Serum levels of progesterone and its metabolites were analyzed, as CYP17A1 metabolizes progesterone to 17-hydroxyprogesterone (17-OHP). In *Akr1b7*^−/−^ mice, serum progesterone levels were significantly elevated, accompanied by a reduction in the 17-OHP/progesterone ratio (Figures 6J and 6K). The increase in the progesterone levels was not detected in mice 6 h after the superovulation (unpublished observation), indicating that the increase was independent of the treatment. Estradiol and testosterone levels did not differ between WT and *Akr1b7*^−/−^ mice (Figure 6K). Consistent with the increase in progesterone levels, the estrous cycle was significantly prolonged to 9.5 days in *Akr1b7*^−/−^ mice (Figures 6L and 6M); primarily due to an extension of the diestrus stage (Figure 6N). The length and timing of estrous cycle stages in *Akr1b7*^*+/−*^ mice were nearly identical to those observed in WT mice (Figures 6M and 6N). NR5A1, an upstream regulator of CYP17A1, has been reported to be activated by phosphatidyl inositol 2-phosphate (PIP2),^23^ and PIP2 produced in theca cell layers of developing follicles and stromal cells was absent in *Akr1b7*^−/−^ mice (Figures 6O, 6P, and S7D). Thus, AKR1B7 regulates the estrous cycle through NR5A1/CYP17A1/progesterone, which may be activated by PIP2 production.

**Figure 7:**
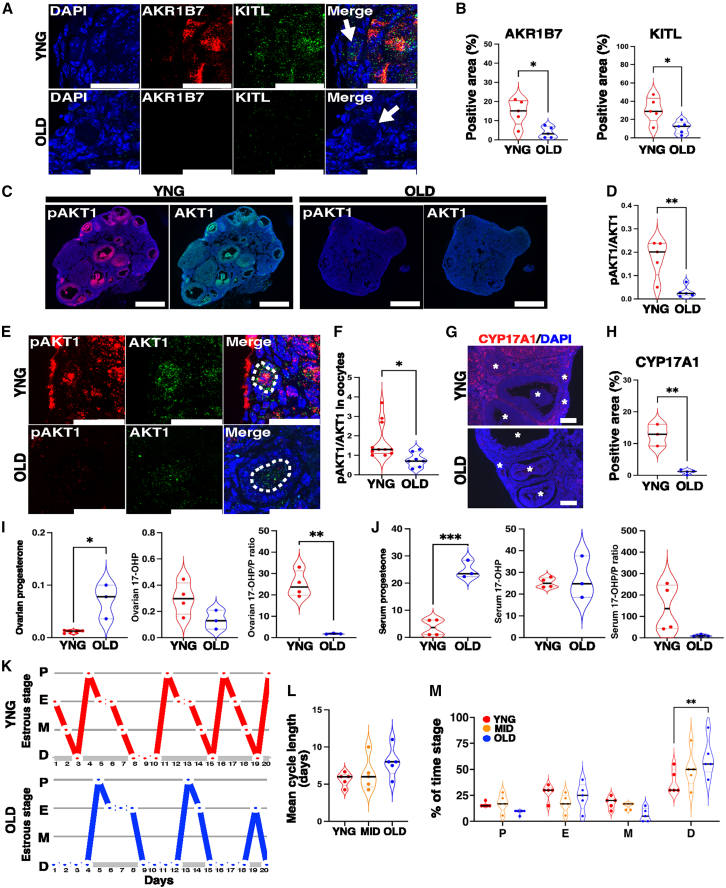
Expression of factors regulated by AKR1B7 in ovarian aging (A–F) Sections were prepared from the ovaries of YNG and OLD mice 24 h after a 5 IU PMSG/hCG injection. (A) Sections stained with anti-AKR1B7 (Cy5, red), anti-KITL (TRITC, green) antibodies, and DAPI (blue). Arrows indicate a primordial follicle. Scale bars, 20 μm. *n* = 5. (B) Violin plots show the positive areas (%) of AKR1B or KITL on tiling images of the whole ovary. *n* = 5, ∗*p* < 0.05; two-tailed Student’s *t* test. (C) Immunofluorescence images show AKT1 and pAKT1 staining in YNG and OLD ovaries. Scale bars, 500 μm. (D) Violin plots show the ratio of pAKT1/AKT1 in (C) *n* = 5, ∗∗*p* < 0.01; two-tailed Student’s *t* test. (E) Sections stained with anti-pAKT (Cy5, red) and anti-AKT antibodies (TRITC, green) and DAPI (blue). Circles with dotted lines indicate the oocyte of a primordial follicle. Scale bars, 20 μm. (F) Violin plots show the ratio of fluorescent intensities of pAKT1 to AKT in the region, as shown in (E) n = 7–11, ∗*p* < 0.05; two-tailed Student’s *t* test. (G) Sections are prepared from the ovaries of YNG and OLD mice 96 h after a 5 IU PMSG/hCG injection and stained with anti-CYP17A1 antibody (Cy5, red) and DAB reagents. Scale bars, 50 μm. (H) Violin plots show the positive areas (%) in (G) n = 3–4, ∗∗*p* < 0.01; two-tailed Student’s *t* test. (I and J) Progesterone and 17-OHP levels, and the ratio of 17-OHP to progesterone, were quantified using the reversed-phase HPLC method. The violin plot shows progesterone and metabolite levels in the ovary (I) or serum (J) from YNG and OLD female mice 96 h after 5 IU PMSG/hCG injection. n = 3–4, ∗*p* < 0.05, ∗∗*p* < 0.01, and ∗∗∗*p* < 0.001; two-tailed Student’s *t* test. (K) Representative estrous cycle patterns, determined by vaginal cytology, are shown for YNG and OLD mice. Proestrus; P, estrus; E, metestrus; M and diestrus; D, *n =* 5. (L) Mean estrous cycle length, determined from (K) and is shown for YNG, MID, and OLD mice. One-way ANOVA followed by the Dunnett’s multiple comparisons test. (M) Violin plots show the percentages of time spent in each stage of the estrous cycle, determined from (K), in YNG, MID, and OLD mice. ∗∗*p* < 0.01; one-way ANOVA followed by the Dunnett’s multiple comparisons test.

During ovarian aging, the transient expression of *Akr1b7* in the theca cell layers of young mice was attenuated, independent of ovulation stimulation. Consistent with AKR1B7 expression, KITL staining was weakly detected only in the stromal cells of aged ovaries, distinct from the apparent staining observed in young ovaries (Figures 7A and 7B). Akt activation during ovarian aging was nearly undetectable throughout the ovary, including in the oocytes of primordial follicles, despite Akt expression (Figures 7C–7F). CYP17A1, which was detected in young ovaries following CG administration, was almost absent in theca cell layers of old ovaries (Figures 7G and 7H). Ovarian progesterone levels were significantly elevated with age, corresponding to a reduction of 17-OHP, a progesterone metabolite produced by CYP17A, as reflected in serum progesterone and 17-OHP levels (Figures 7I, 7J, and S8). Consistent with sustained progesterone levels, the estrous cycle was prolonged due to an extended diestrus stage in the old mice (Figures 7K–7M). This cycle prolongation was not observed in the middle-aged 24-week-old mice (Figures 7L and 7M). The signaling pathways attenuated in aged mice were similar to those observed in *Akr1b7*^−/−^ mice.

## Discussion

Our data revealed genotype-specific phenotypes that evolved distinctly with age, highlighting a gene-age interaction in ovarian function. At 8 weeks, *Akr1b7*^−/−^ mice exhibited normal follicular morphology and ovulation parameters comparable to those of WT and *Akr1b7*^*+/−*^ mice, indicating a preserved ovarian reserve during early reproductive life. By 16 weeks, however, *Akr1b7*^−/−^ mice developed reproductive defects, including increased ovulation of immature oocytes, reduced mitochondrial membrane potential, decreased numbers of primordial follicles, and smaller litters, features typically observed in 48-week-old WT mice. In contrast, *Akr1b7*^*+/−*^ mice maintained a reproductive profile similar to that of WT animals at all examined time points (8 and 16 weeks), with normal litter sizes, oocyte maturation rates, and estrous cycle lengths. These findings suggest that *Akr1b7*^*+/−*^ mice retain sufficient gene dosage to support both folliculogenesis and endocrine function. At 16 weeks, both WT and *Akr1b7*^*+/−*^ mice exhibited stable reproductive parameters, whereas *Akr1b7*^−/−^ mice displayed a persistent functional decline. By 48 weeks, WT mice naturally recapitulated the *Akr1b7*^−/−^ phenotype, indicating that complete loss of *Akr1b7* lowers the threshold for reproductive dysfunction under aging-related stressors. This age-dependent penetrance underscores the critical role of *Akr1b7* in preserving ovarian integrity and preventing premature reproductive senescence. To assess cellular stress and senescence-associated features associated with *Akr1b7* loss, we evaluated senescence-associated β-galactosidase (β-gal) activity, which was elevated in *Akr1b7*^−/−^ ovaries. Along with elevated 8-OHdG levels—an oxidative stress (DNA damage) marker—these findings indicate that *Akr1b7* deficiency is associated with impaired oocyte maturation and increased oxidative stress within ovarian tissue. Importantly, 8-OHdG serves as an oxidative stress (DNA damage) indicator rather than a chronological or functional aging marker; its elevation reflects increased oxidative burden in aged or *Akr1b7*-deficient ovaries. While impaired oocyte maturation arises directly from endocrine dysregulation in theca cells, the broader phenotype likely reflects premature ovarian aging driven by increased oxidative stress and reduced cellular resilience in the absence of *Akr1b7*.

A decline in both the quantity and quality of oocytes is a hallmark of ovarian aging, particularly associated with the developmental failure of early-stage follicles, such as primordial follicles. Ovarian aging manifests as reproductive dysfunction, including extended menstrual cycles and hormonal abnormalities.^2^ In a mouse model of ovarian aging, the number of follicles and CLs was significantly lower at 48 weeks than at 8 weeks, although the follicles remained in the ovaries of 48-week-old mice.^24^ The time-course analysis following hCG stimulation was designed to evaluate the dynamics of follicular responsiveness rather than full maturation, as development from primordial follicle activation to ovulation requires several weeks in mice. The reduced depletion of early-stage follicles in aged mice following gonadotropin stimulation suggests a failure to appropriately recruit and advance follicles toward ovulation. This likely reflects the dysregulation of follicular activation thresholds or hormonal signaling pathways in the aged ovary. Furthermore, the overall reduction in follicle numbers at all stages, even before stimulation, supports the well-established observation of diminished ovarian reserve with age. Based on these findings, 48-week-old mice were used as a model of ovarian aging. Pathway analysis demonstrated a reduction in the LH/CG-regulated AKR1B7 pathway, accompanied by the downregulation of genes related to glucose and lipid metabolism in the aging mouse model. Ge et al. reported that AKR1B7 regulates lipid and glucose homeostasis in the liver,^25^ indicating that decreased *Akr1b7* expression may impact downstream pathways, such as glucose and lipid metabolism. *Akr1b7*^−/−^ mice produced using i-GONAD exhibited accelerated ovulation of immature oocytes with relatively small diameters, resulting in reduced litter size. The ovulation of immature oocytes observed in *Akr1b7*^−/−^ ovaries may be mediated by Akt activation for the following reasons. First, Akt activation, which is essential for follicular development in primordial follicles in response to FSH,^26^ was absent in the oocytes of primordial follicles in *Akr1b7*^−/−^ mice. Second, *Akt*^−/−^ ovaries treated with exogenous gonadotropins exhibited a reduction in primordial follicles and an increase in secondary follicles,^27^ as well as a delayed onset of the estrous cycle,^28^ closely mirroring the phenotypes observed in *Akr1b7*^−/−^ mice. Interestingly, the reduced primordial follicle count in *Akr1b7*-deficient mice occurs despite the suppression of the KITL-KIT-pAKT signaling axis, which normally promotes primordial follicle activation. Conventionally, inhibition of this pathway would be expected to delay follicle activation and preserve the dormant pool. However, our findings, along with previous reports on *Akt*-deficient mice, suggest otherwise. Consistently, *Akr1b7*^−/−^ ovaries exhibit suppressed KITL expression in stromal cells and impaired pAKT activation in oocytes of primordial follicles. These results support the notion that AKR1B7 is necessary for both follicle activation and the maintenance of oocyte viability during early folliculogenesis. Therefore, the reduction in primordial follicles in *Akr1b7*-deficient mice likely reflects compromised oocyte integrity rather than excessive activation, contributing to the premature onset of ovarian aging phenotypes. Finally, Akt inactivation is associated with glucose metabolism disorders linked to diabetes, as observed in *Akr1b7*^−/−^ mice.^29^ Ovarian glucose metabolism is closely linked to the PI3K/Akt signaling pathway, which contributes to folliculogenesis dysfunction, as observed in ovarian aging.^27^

How does AKR1B7, present in stromal cells, activate Akt in the oocyte of primordial follicles? Human AKR1B10, an ortholog of mouse AKR1B7 (Alliance of Genome Resources, Apr 2022), binds to acetyl-CoA carboxylase (ACC), a rate-limiting enzyme in fatty acid synthesis, thereby preventing the ACC degradation.^30^ AKR1B7 deletion may lead to ACC degradation due to its dissociation, similar to the effect seen with AKR1B10. As shown in Figure 5E, pathway analysis of *Akr1b7*^−/−^ mouse ovaries revealed the decreased concentrations of lipids and triacylglycerol, indicating the downregulation of lipid synthesis. Reduced ACC levels trigger a metabolic switch toward glycolysis due to the inhibition of acetyl-CoA conversion to malonyl-CoA.^31^ Inhibition of mitochondrial respiration increases AMP/ADP levels, leading to AMPK activation.^32^ AMPK activation may inactivate Akt by decreasing KITL levels, whereas AMPK inhibition triggers KITL release, thereby promoting Akt activation in oocytes of primordial follicles.^33^ In the ovary, KITL staining was observed in the cytoplasm of *Akr1b7*-expressed stromal cells, in addition to the membrane staining of granulosa cells, suggesting that *Akr1b7* may regulate cytoplasmic KITL2 expression, distinct from the membrane-bound KITL1 in granulosa cells. Consistent with our findings, human KITL expression has been observed in both granulosa and stromal cells.^34^ Introduction of KITL restored fertility in fertile mice.^35^ Reduced KITL expression in the stromal cells of *Akr1b7*^−/−^ mice may be associated with primordial follicle arrest.

The network pathways activated over the time course following ovulation stimulation differed between the young and old mice. In the ovaries of young mice, estrogen and its receptors activated the gonadotropin-releasing hormones *Gnrh2* and *Pgh2*, which were associated with hCG activation 48 h after ovulation stimulation.^36^^,^^37^ In the terminal phase of the cycle, the CG, FSH, and LH pathways induced the expression of metabolic enzymes involved in sex hormone synthesis, such as *Cyp17a1* and *Hsd17b3*.^38^^,^^39^ In the aged ovaries, major pathways activated by sex hormones, excluding exogenous LH and FSH, were not detected until 96 h after stimulation. Gene markers associated with macrophages and T cells, such as NADPH oxidase, IL4, TCR, and CD3, are present in these pathways. Previous studies have indicated that inflammation and oxidative stress are closely associated with ovarian aging.^14^^,^^24^^,^^40^ During the formation of the secondary follicles, theca cells are recruited through differentiation from progenitor cells in the stromal compartment.^41^ Theca cell marker genes, including *Cyp17a1, Col1a2*, and *InsI3* mRNA, were significantly decreased in the old mice,^42^ indicating that the differentiation of stromal cells into theca cells may be defective in aged ovaries. Previous studies have shown that theca cells contribute to steroidogenesis, which is necessary for folliculogenesis and the estrous cycle.^43^ As demonstrated in this study, *Akr1b7* was localized to theca cells and stromal cells. Previous reports indicate that the major activities of AKR1B7 include the detoxification of steroid metabolites and the synthesis of PGF_2α_.^44^^,^^45^ The activity of toxic metabolite detoxification in *Akr1b7*^−/−^ ovaries was comparable to that in WT mice, although it was reduced in old mice with decreased *Akr1b7* expression. Some AKR1B7 functions in *Akr1b7*^−/−^ mice may be compensated by AKR1B8, which shares high homology with *Akr1b7*, as *Akr1b8* mRNA levels were maintained in *Akr1b7*^−/−^ mice but declined in old mice. AKR1B8 possesses enzymatic activities for ICA and 4-HNE,^44^ but may be limited in compensating for all AKR1B7 functions in theca cells, as its substrate specificity differs from AKR1B7 expression, except for ICA and 4-HNE.^46^ The metabolic pathways involved in sex hormone production are activated in theca cells, and sex hormones strictly regulate the estrous cycle.^2^ Compared with the WT mice, the *Akr1b*7^−/−^ mice exhibited a prolonged estrous cycle. Progesterone levels remained elevated, consistent with a previous report on mice lacking exons 2−4 of *Akr1b7*, yet their litter sizes were nearly comparable to those of WT mice up to 5 months of age.^16^ It is possible that exon 1 in *Akr1b7* plays a role in oocyte maturation, as mice with a deletion starting from the *Akr1b7* codon exhibited reduced litter sizes and an increased number of immature oocytes at 16 weeks of age. Although a modest reduction in primordial follicle numbers was observed in 16-week-old *Akr1b7*^−/−^ mice, this decline occurred without a corresponding loss of developing follicles, distinguishing it from the global follicle depletion characteristic of advanced ovarian aging in 48-week-old mice. We propose that this early decrease in the dormant follicle pool may represent an initiating step toward accelerated ovarian aging, eventually leading to more pronounced follicular attrition and reproductive decline with advancing age.

The litter size of 8-week-old *Akr1b7*^−/−^ mice was comparable to that of age-matched WT mice, consistent with our findings. However, prolonging breeding to 16 weeks of age resulted in a significant reduction in litter size, accompanied by an increase in immature ovulated oocytes in *Akr1b7*^−/−^ mice, suggesting that age-related factors may contribute to the decline in litter size. The MII (PB1 extrusion) rate observed in young ICR females (∼65%) in our study falls within the normal range reported for comparable superovulation protocols, although it is somewhat higher or lower than specific prior datasets depending on experimental parameters. Miao et al.^47^ reported ∼85% MII oocytes in 6–8-week-old ICR mice following 5 IU PMSG and 10 IU hCG, with oocytes collected at13.5 h post-hCG, whereas lower rates of 46–51% were reported for 2–3-month-old CD-1 (ICR) mice using 5 IU PMSG and 5 IU hCG with a 14 h collection interval.^48^ Similarly, Golkar-Narenji et al. reported 45–65% MII rates in BALB/c and NMRI strains under 7.5 IU PMSG/hCG stimulation at 16 h.^49^ Our stimulation protocol (5 IU PMSG +5 IU hCG, with oocyte collection at 16 h after hCG injection) and mouse age (6–8 weeks, ICR strain), therefore, produced values consistent with inter-laboratory variability. Such differences are known to reflect strain background, hormonal dose and source, animal age, and housing or colony-specific variation inherent to closed-colony ICR mice. The increase in the progesterone levels was accompanied by a reduction in 17-OH-metabolites, consistent with the decreased expression of *Cyp17a1* localized in theca cells. *Cyp17a1* expression is regulated by NR5A1/SF-1,^50^ whose target genes exhibited reduced expression in *Akr1b7*^−/−^ mice, despite NR5A1/SF-1 expression and localization remaining unchanged. This suggests that the ligand is suppressed in KO mice. NR5A1/SF-1 directly binds to PIP2, which is converted to PIP3 and functions as a ligand for NR5A1/SF-1.^51^^,^^52^ Exposure to gonadotropins induced PIP2 formation in the theca cell layers and at the boundary regions of the granulosa cell layers; however, this PIP2 product was eliminated in KO mice, as shown in Figure 6O. In a breast cancer cell line transfected with siRNA targeting *Akr1b10*, a human ortholog of *Akr1b7*, PIP2 production was significantly reduced, leading to decreased cell growth. Several studies have implicated phospholipid metabolism in the AKR superfamily. AKR1B10 regulates the stability of acetyl-Co carboxylase, which may indirectly contribute to the synthesis of membrane lipids, such as PIP2.^53^ Spite et al. reported that the AKR1B superfamily exhibits enzymatic activity against phospholipid aldehydes present in the PIP2 structure,^54^ which may be directly metabolized by AKR1B7 as a substrate. The reduction in phospholipids by AKRs could modulate signaling mechanisms triggered by biologically active phospholipids, including those containing aldehyde structures.^55^ PIP2 generated by AKR1B7 regulates the estrous cycle through the progesterone/CYP17A1/NR5A1 pathway and may also influence the normal development of primordial follicles through Akt activation.

In conclusion, *Akr1b7*, expressed in theca and stromal cells of the ovary, induces KITL expression in response to ovulation stimulation, thereby promoting follicular development through Akt activation. In addition, *Akr1b7* regulates the estrous cycle through progesterone metabolism via *Cyp17a1* expression. Reduced *Akr1b7* expression was associated with the ovulation of immature oocytes and prolonged estrous cyclicity, phenotypes that overlap with features observed during reproductive aging. Additional studies will be required to determine whether *Akr1b7* downregulation is causal for age-associated reproductive decline or reflects broader aging-related changes.

Although the present study demonstrates that *Akr1b7* deficiency is associated with impaired oocyte maturation and altered endocrine profiles, we did not include formal litter-based fertility testing. Fertility assessment was considered beyond the mechanistic scope of this work, as reproductive outcomes in a conventional knockout may be influenced by extra-ovarian factors, including pituitary or adrenal contributions. Accordingly, the interpretation of fertility endpoints would not isolate ovarian-specific mechanisms. Ongoing studies using ovarian cell-specific *Akr1b7* deletion and rescue models aim to directly address reproductive outcomes and clarify tissue-specific functions of AKR1B7 in fertility regulation.

### Limitations of the study

Because this study used a conventional *Akr1b7* knockout, it cannot distinguish between ovarian and extra-ovarian contributions (e.g., pituitary or adrenal effects). Cell-autonomous mechanisms will require ovarian cell-specific deletion or rescue approaches.

## Resource availability

### Lead contact

Further information and requests for resources and reagents should be directed to and will be fulfilled by the Lead contact, Yoichi Mizukami (mizukami@yamaguchi-u.ac.jp).

### Materials availability

All unique reagents generated in this study are available from the lead contact upon reasonable request.

### Data and code availability

• Data: RNA-seq and SuperSAGE data generated in this study have been deposited at GEO under accession numbers GSE238259 and GSE238260, and are publicly available as of the publication date. Additional information necessary to reanalyze the data reported in this paper is available from the lead contact upon request.
• Code: This paper does not report original code.
• Other: Any additional information required to reanalyze the data reported in this paper is available from the lead contact upon request.

## Acknowledgments

This work was supported by research equipment shared through the 10.13039/501100001700MEXT Project for promoting the public utilization of advanced research infrastructure (program for supporting the construction of core facilities; grant number JPMXS0440400021-23) and grants-in-aid for scientific research (grant numbers: 20K17491, 21K08644, 21H03358, and 22K19711) from the 10.13039/501100001700Ministry of Education, Culture, Sports, Science and Technology of Japan.

We thank Ms. Kaori Kaminoyama (Center for Plant Sciences, 10.13039/100022859Kyoto Sangyo University) for conducting the next-generation sequencing (NGS) analysis using the NextSeq500. We also thank Ms. Makiko Nakagawa, Ms. Takako Moriyama, and Ms. Yuko Nakatani (10.13039/100016983Yamaguchi University) for their technical assistance with the NGS analysis. We also appreciate the technical support provided at the 10.13039/100016983Yamaguchi University Science Research Center.

## Author contributions

Conceptualization, K.I. and Y.M.; methodology, K.I., K.W., M.O., S.K., T.H., S.S., and Y.M.; investigation, K.I. and K.W.; writing, K.I. and Y.M.; funding acquisition, K.I., K.W., and Y.M.; supervision, T.M., M.A., H.T., N.S., and Y.M.

## Declaration of interests

The authors declare no competing interests.

## Declaration of generative AI and AI-assisted technologies in the writing process

During the preparation of this work, the authors used the ChatGPT-5.0 model in order to assist in improving the clarity and accuracy of the language. After using this tool, the authors reviewed and edited the content as needed and take full responsibility for the content of the published article.

## STAR★Methods

### Key resources table

**Table:** 

REAGENT or RESOURCE	SOURCE	IDENTIFIER
**Antibodies**		

Rabbit polyclonal anti-AKR1B10	Thermo Fisher Scientific	Catalog #PA5-22036; RRID: AB_11152818
Mouse monoclonal anti-DNA/RNA damage (8-OHdG)	Abcam	Catalog #ab62623; RRID: AB_940049
Rabbit monoclonal anti-CYP17A1	Abcam	Catalog # ab125022; RRID: AB_10975095
Rabbit monoclonal anti-NR5A1	Cell Signaling Technology	Catalog #12800S; RRID: AB_2798030
Rabbit monoclonal anti-AKT1	Cell Signaling Technology	Catalog #75692; RRID: AB_2716309
Rabbit monoclonal anti-pAKT1	Abcam	Catalog #ab81283; RRID: AB_2224551
Rabbit monoclonal anti-AKT (pan)	Cell Signaling Technology	Catalog #4691; RRID: AB_2100659
Mouse monoclonal anti-PI(4,5)P2	Echelon biosciences	Catalog #Z-P045; RRID: AB_427225
Rabbit polyclonal anti-KITL/SCF	Abcam	Catalog #ab64677; RRID: AB_1861346
anti-GAPDH monoclonal antibody conjugated peroxidase	FUJIFILM Wako Pure Chemical	Catalog # 015-25473; RRID: AB_2665526
Omni-Map anti-rabbit HRP	Roche Diagnostics	Catalog # 760-4311; RRID: AB_2811043
Omni-Map anti-mouse HRP	Roche Diagnostics	Catalog # 760-4310; RRID: AB_2885182
Anti-rabbit IgG conjugated peroxidase	New England Biolabs	Catalog # 7071-1; RRID: N/A
Goat F(ab) anti-mouse IgG H&L	Abcam	Catalog # ab6668; RRID: 955960

**Chemicals, peptides, and recombinant proteins**		

CAS9 protein: Alt-R® S.p. Cas9 Nuclease V3	Integrated DNA Technologies	Catalog # 1081058
Opti-MEM™I without phenol red	Thermo Fisher Scientific	Catalog # 11058021
mHTF medium	KYUDO CO.	N/A
Hyaluronidase	Sigma-Aldrich	Catalog #H3506
PMSG (product name: SEROTROPIN ®)	ASKA Animal Health	N/A
hCG (product name: Gonadotropin for animal)	ASKA Animal Health	N/A
Protease inhibitor cocktail	Sigma-Aldrich	Catalog #P8340
Phosphatase inhibitor cocktail3	Sigma-Aldrich	Catalog #P0044
Indomethacin	Sigma-Aldrich	Catalog #I7378
Isocaproaldehyde	Santa Cruz	Catalog # sc-483809
4-Hydroxynonenal	Santa Cruz	Catalog # sc-202019
NADH	Sigma-Aldrich	Catalog #N8129
Progesterone	FUJIFILM Wako Pure Chemical	Catalog # 160-24511
17α-hydroxy progesterone	Tokyo Chemical Industry	Catalog #H1250
20α-hydroxy progesterone	Santa Cruz	Catalog # sc-396005
Nandrolone	Tokyo Chemical Industry	Catalog #N0777
RNA later®	Thermo Fisher Scientific	Catalog # AM7021
EnVision™ FLEX Target antigen retrieval solution, high pH	Agilent	Catalog #K8044
Acetonitrile for HPLC	FUJIFILM Wako Pure Chemical	Catalog # 015-08633
Ethyl acetate	FUJIFILM Wako Pure Chemical	Catalog # 051-00356
Diethyl ether	FUJIFILM Wako Pure Chemical	Catalog # 055-01155
Tris(hydroxymethyl)aminomethane	Nacalai tesque	Catalog # 35434-21
Hydrochloric acid	FUJIFILM Wako Pure Chemical	Catalog # 080-01066
Phosphate-Buffered Saline	FUJIFILM Wako Pure Chemical	Catalog # 163-25265
Sucrose	FUJIFILM Wako Pure Chemical	Catalog # 196-00015
Giemsa’s stain solution	Nacalai tesque	Catalog # 37114-64
Fetal Bovine Serum	Biowest	Catalog #S1650
Random primer 9	New England Biolabs	Catalog #S1254S
RNase inhibitor	Takara	Catalog # 2311A
M-MuLV reverese transcriptase	New England Biolabs	Catalog #M0253S
0.1% poly-L-lysine solution in H_2_O	Sigma-Aldrich	Catalog #P8920
Paraformaldehyde	FUJIFILM Wako Pure Chemical	Catalog # 160-16061
VECTASHIELD with DAPI	Vector laboratories	Catalog # H-1200
Xylene	Nacalai tesque	Catalog # 36611-45
Mayer’s hematoxylin solution	FUJIFILM Wako Pure Chemical	Catalog # 131-09665
0.5% eosin Y solution	Merck	Catalog#1.09844.1000
O.C.T compound	Sakura-finetek	Catalog # 4583
Bovine Serum Albumin	Nacalai tesque	Catalog # 01863-48
Triton X-100	FUJIFILM Wako Pure Chemical	Catalog # 595-13135
CC1 buffer (EDTA-based buffer) for VENTANA	Roche Diagnostics	Catalog # 518-108939
CC2 buffer (Citrate-based buffer) for VENTANA	Roche Diagnostics	Catalog # 518-108946
Discovery ULTRA Cy5 for VENTANA	Roche Diagnostics	Catalog # 760-238
Discovery ULTRA Rhodamine for VENTANA	Roche Diagnostics	Catalog # 760-233
Discovery ULTRA FITC for VENTANA	Roche Diagnostics	Catalog # 760-232
SPiDER-betaGal	DOJINDO	Catalog # SG02

**Critical commercial assays**		

RNeasy Mini Kit	QIAGEN	Catalog # 74104
RNeasy FFPE Kit	QIAGEN	Catalog # 73504
QuantiTect SYBR Green PCR Kit	QIAGEN	Catalog # 204143
SOLiD™SAGE™ Kit	Thermo Fisher Scientific	Catalog # 4452811
SOLiD™ RNA Barcoding Kit	Thermo Fisher Scientific	Catalog # 4452811
Pure-Link PCR Micro Kit	Thermo Fisher Scientific	Catalog #K310050
NEBNext Ultra II RNA library Prep kit for Illumina	New England Biolabs	Catalog #E7770
NextSeq 500/550 High Output kit v2.5 (150 cycles)	Illumina	Catalog # 20024907
PGF2α ELISA kit	Cayman chemical	Catalog # 516011
Estradiol ELISA kit	Cayman chemical	Catalog # 501890
Testosterone ELISA kit	Cayman chemical	Catalog # 582701
Progesterone ELISA kit	Cayman chemical	Catalog # 582601
Protein Assay Dye Reagent Concentrate	Bio-rad	Catalog # 5000006
N-Histofine® Simple Stain Mouse MAX PO	Nichirei bioscience	Catalog # 424131
Mouse on mouse polymer IHC kit	Abcam	Catalog # ab269452
Mouse and Rabbit Specific HRP/DAB IHC Detection Kit	Abcam	Catalog # ab236466
Vector TrueVIEW Autofluorescence Quenching Kit with DAPI	Vector laboratories	Catalog # SP-8500-15
ChromoMap DAB kit	Roche Diagnostics	Catalog # 760-159
Deposited Data		
RNA seq data	This paper	GSE238259
SuperSAGE data	This paper	GSE238260

**Experimental models: Organisms/strains**		

Mouse: ICR	Japan SLC,Inc	N/A
Mouse: ICR-*Akr1b7*^em1Miz^	This paper	N/A
Mouse: C57BL6/N	Japan SLC,Inc	N/A

**Oligonucleotides**		

Guide RNA targeting sequence: TTTGGTACTGAGTTCCACGA	Integrated Dna Technologies	Design ID: Mm.Cas9.AKR1B7.1.AN
qPCR Primers and TaqMan probes, see Table S1	FASMAC	N/A

**Software and algorithms**		

XSQ-Tool	Thermo Fisher Scientific	http://solidsoftwaretools.com/gf/project/xsq/
bcl2fastaq Conversion Software v2.19	Illumina	https://jp.support.illumina.com/sequencing/sequencing_software/bcl2fastq-conversion-software/downloads.html
Real Time Analysis	Illumina	https://jp.support.illumina.com/sequencing/sequencing_software/real-time_analysis_rta/downloads.html
CLC Genomics Workbench	QIAGEN	https://digitalinsights.qiagen.com/products/qiagen-clc-genomics-workbench/
Prism 9	GraphPad	https://www.graphpad.com/scientific-software/prism/
JMP pro14	SAS institute	https://www.jmp.com/
Ingenuity Pathway Analysis	QIAGEN	https://digitalinsights.qiagen.com/products/qiagen-ipa/
ImageJ	NIH	https://imagej.nih.gov/ij/
MetaMorph imaging software	Molecular devices	https://www.moleculardevices.com/products/cellular-imaging-systems/acquisition-and-analysis-software/metamorph-microscopy/
Empower 3 software	Waters	https://www.waters.com/waters/en_US/Empower-Chromatography-Data-System
ImageQuant TL software	Cytiva	https://www.cytivalifesciences.co.jp/catalog/1167.html
Las X	Leica microsystems	http://www.leica-microsystems.com/products/microscope-software/details/product/leica-las-x-ls/
BZ-X800 analyzer	Keyence	https://www.keyence.co.jp/products/microscope/fluorescence-microscope/bz-x700/models/bz-x800/
Imaris	Oxford instruments	https://imaris.oxinst.com/products/imaris-for-cell-biologists

### Experimental model and study participant details

#### Animals and the collection of ovaries

Female C57BL/6N mice at the age of 6–10 weeks (“YNG”), 24 weeks (“MID”), and 48–56 weeks (“OLD”) were purchased from Japan SLC Inc (Shizuoka, Japan). To synchronize the estrous cycle, we performed a superovulation procedure by administering pregnant mare serum gonadotropin (PMSG, 5 IU; ASKA Pharmaceutical, Tokyo, Japan) and human chorionic gonadotropin (hCG, 5 IU; ASKA Pharmaceutical) at 48 h intervals to mice. Mice were euthanized 24, 48, 72, and 96 h after hCG administration. Ovaries were collected at indicated time points within 5–10 min after euthanasia by CO_2_ inhalation. One side of the ovary was quickly frozen in liquid nitrogen and stored in RNAlater (Thermo Fisher Scientific, Waltham, MA) at −80°C for the expression analyses. The other side was fixed with 4% paraformaldehyde and embedded in paraffin for histological analysis. ICR mice (Japan SLC Inc.) were used to generate gene-edited mice and maintained in an ICR genetic background. Female wild-type (WT), *Akr1b7*^+/−^, and *Akr1b7*^−/−^ ICR mice were examined at 6–8 weeks (“YNG”), or 16 weeks of age. Male WT or *Akr1b7*^+/−^ ICR mice aged 3–5 months old were used for mating. The primers and TaqMan probes used for genotyping are listed in Table S1. Females were group-housed with up to 5 mice per cage under a stable temperature in a 12 h light and dark cycle with water and food *ad libitum*. All experiments were performed in accordance with the recommendations of the Guide for Animal Experiments at the Yamaguchi University School of Medicine. The Committee on the Ethics of Animal Experiments of Yamaguchi University School of Medicine reviewed and approved all procedures. All experiments were conducted in females due to the ovary-specific nature of the study, and gender differences were not assessed.

### Method details

#### Histochemical analysis

Pretreatment of the tissues was performed as previously described.^56^ The fixed ovaries with 4% paraformaldehyde were embedded in paraffin block and serially sectioned at 8 μm using a microtome. The sections were stained with hematoxylin and eosin (H&E). Classification and counting of ovarian follicles at different stages were performed as previously described.^57^

#### Follicle counts

Follicles were classified according to established morphological criteria: the follicles with oocyte surrounded by a single layer of squamous granulosa cells were characterized as primordial follicles, the follicles with oocyte surrounded by a single layer of cuboidal granulosa cells were characterized as primary follicles, the follicles with oocyte surrounded by multiple layers of cuboidal granulosa cells with or without antral space development were characterized as secondary follicles, and the follicles with oocyte situated on cumulus oophorus with multiple layers of granulosa cells and a large confluent antral space were characterized as antral follicles. The total number of primordial and primary follicles was counted in every fifth section of the ovary using a method of direct counts.^58^ Because the minimum diameters for primordial and primary follicles were 7–25 μm,^59^ smaller than 40 μm, corresponding to an 8 μm x fifth section interval, raw counts were multiplied by 5 to obtain the estimated number. The total number of secondary and antral follicles was counted using the raw number for each fifth section. Repetitive counting was avoided by counting only follicles containing an oocyte with a visible nucleolus. The number of CLs, including ovulated luteinizing follicles, was counted using the raw number to avoid repetition among serial sections. All ovaries used for comparison were processed under identical conditions and evaluated using the same counting rules. Because absolute follicle numbers are known to vary depending on fixation type, embedding material, section thickness, sampling fraction, and counting approach (direct counts vs. stereology),^58^ we adopted a standard fixation and embedding procedure consistent with the previous report. Total follicle counts obtained in this study align with those reported in similar datasets using conventional fixation methods. All statistical analyses were performed under blinded conditions as within-study, like-for-like comparisons rather than as absolute cross-study values. Data are presented as relative differences with respect to control values.

#### Generation of *Akr1b7*-deficient mice using i-GONAD-mediated genome editing

i-GONAD was performed as previously described.^60^^,^^61^ In brief, surgical procedures were performed on anesthetized ICR females on pregnant day 0.7 under observation by using a dissecting microscope (SZ60; Olympus, Tokyo, Japan). The reproductive tract (ovary-oviduct-uterus) was gently pulled out of the abdominal cavity, and the position was fixed by holding the fat pad with an artery clip. Moreover, 1.5 μL per an oviduct of Opti-MEM (Thermo Fisher Scientific) containing 30 μM annealed *Akr1b7*-crRNA/tracrRNA (Integrated DNA Technologies, Coralville, IA) and 1 mg/μL CAS9 protein (Integrated DNA Technologies) was injected into the oviductal lumen from upstream of the ampulla by using a micropipette connected to a mouthpiece (Kitazato corp, Shizuoka, Japan). The oviductal ampulla filled with CRISPR/CAS9 solution was covered with a piece of wet paper (Kimewipe, Japan) soaked in PBS and grabbed with a tweezer-type electrode. Electroporation was performed using CUY21EDIT-II (BEX, Tokyo, Japan) under the following parameters: square (mA), Pd V:80 V, Pd A:150 mA, Pd on:5.00 ms, Pd off:50 ms, Pd N:3, Decay:10%, and Decay-Type: Log (+/−). After the surgical procedure, the mice were incubated on a heating pad at 37°C until they awoke from the anesthesia.

#### Monitoring of estrous cycle

Vaginal smears on a microscope slide were stained with Giemsa’s stain solution (Nacalai Tesque, Kyoto, Japan), as previously described,^62^ and classified into 1 of 4 estrous stages, as previously described: proestrus, estrus, metestrus, and diestrus.^63^ The mean cycle length represents the mean of the days between the estrus and the next estrous cycle.

#### Analysis of the ovulated oocytes

Mice were treated by administering 5 IU PMSG and 5 IU hCG at 48 h intervals. Sixteen hours after hCG administration, mice were euthanized by CO_2_ inhalation, and ovulated oocytes were collected from the oviductal ampullae on both sides. Oocytes were incubated in mHTF medium (Kyudo, Tosu, Japan) containing 0.1 mg/mL hyaluronidase enzyme (Sigma-Aldrich, *St. Louis, MO*) for 5 min at 37°C to remove cumulus cells. Due to the movement of the oocytes within the culture medium, imaging multiple oocytes reliably within a single microscopic field was not feasible. To ensure accurate evaluation, oocytes from each mouse were transferred into individual wells of a 24-well culture plate. Tiling images of each well were captured using a 10x phase-contrast objective lens on an all-in-one BZ-X800 microscope (Keyence, Osaka, Japan). The number and maturation status of oocytes were assessed in all images. Oocytes lacking a first polar body at the germinal vesicle stage or in metaphase I, as well as degenerated oocytes, were considered immature, whereas those with a first polar body at metaphase II were considered mature, as previously described.^64^ The average diameter of an oocyte, including the zona pellucida, was calculated from the value of the major and minor axes using the automatic-measuring application of the BZ-X800. Measurements with an average diameter outside the range of 75 μm–135 μm were excluded to eliminate inaccuracies. To assess mitochondrial membrane potential, ovulated oocytes were washed with M2 medium (Sigma-Aldrich) and incubated in M2 medium containing 2.5 μg/mL JC-1 (Thermo Fisher Scientific) at 37°C in 5% CO_2_ for 30 min. After washing, green fluorescence emission at 525 nm and red fluorescence emission at 590 nm were captured and analyzed using a CQ1 confocal microscope (Yokogawa, Tokyo, Japan).

#### Analysis of the litter size

WT, *Akr1b7*^*+/−*^ and *Akr1b7*^−/−^ females underwent a superovulation procedure by administering 5 IU PMSG followed by 5 IU hCG at 48 h intervals. These females were then bred with WT males. For natural mating, WT, *Akr1b7*^*+/−*^*,* and *Akr1b7*^−/−^ females were bred without any treatment. The number of offspring was recorded for females in which a vaginal plug was confirmed.

#### Senescence-associated β-galactosidase staining

Ovaries were embedded in Tissue-Tek OCT compound (Sakura Finetek, Tokyo, Japan), rapidly frozen, and sectioned at a thickness of 10 μm. Following the manufacturer’s protocol, sections were fixed in 4% paraformaldehyde for 20 min at room temperature and then washed with PBS. Samples were incubated with 20 μmol/L SPiDER-βGal (DOJINDO, Kumamoto, Japan) at 37°C for 30 min, followed by washing with PBS and imaging using an all-in-one fluorescence microscope, BZ-X800 (Keyence).

### Quantification and statistical analysis

#### Statistics and reproducibility

Statistical analyses were performed using GraphPad Prism version 9. Student’s two-tailed t tests were used for statistical comparisons of data obtained under the 2 conditions. One-way analysis of variance (ANOVA) followed by Tukey’s multiple comparison test or Dunnett’s multiple comparison test was used for the statistical comparison of more than 2 groups. All data are expressed as the means ± SE. Statistical significance was set at *p* < 0.05. Sample sizes and numbers are indicated in detail in the legends of each figure.

## Supplemental information

Document S1. Figures S1–S8 and Methods S1

Table S1. Sequence of qPCR-primers, TaqMan probes, and guide RNA
