# Supplementary material for: Reduced Akr1b7 signaling drives ovarian aging and reproductive dysfunction
Source: iScience. 2026 Jan 19;29(2):114719. doi: 10.1016/j.isci.2026.114719 (PMC12918200; doi:10.1016/j.isci.2026.114719)
Supplement: Document S1. Figures S1–S8 and Methods S1 [file mmc1.pdf]

iScience, Volume *29*

Supplemental information

Reduced *Akr1b7* signaling drives ovarian aging and reproductive dysfunction

Keishiro Isayama, Kenji Watanabe, Masato Ohtsuka, Seisuke Kimura, Tomoaki Murata, Takeshi Honda, Masataka Asagiri, Shun Sato, Hiroshi Tamura, Norihiro Sugino, and Yoichi Mizukami

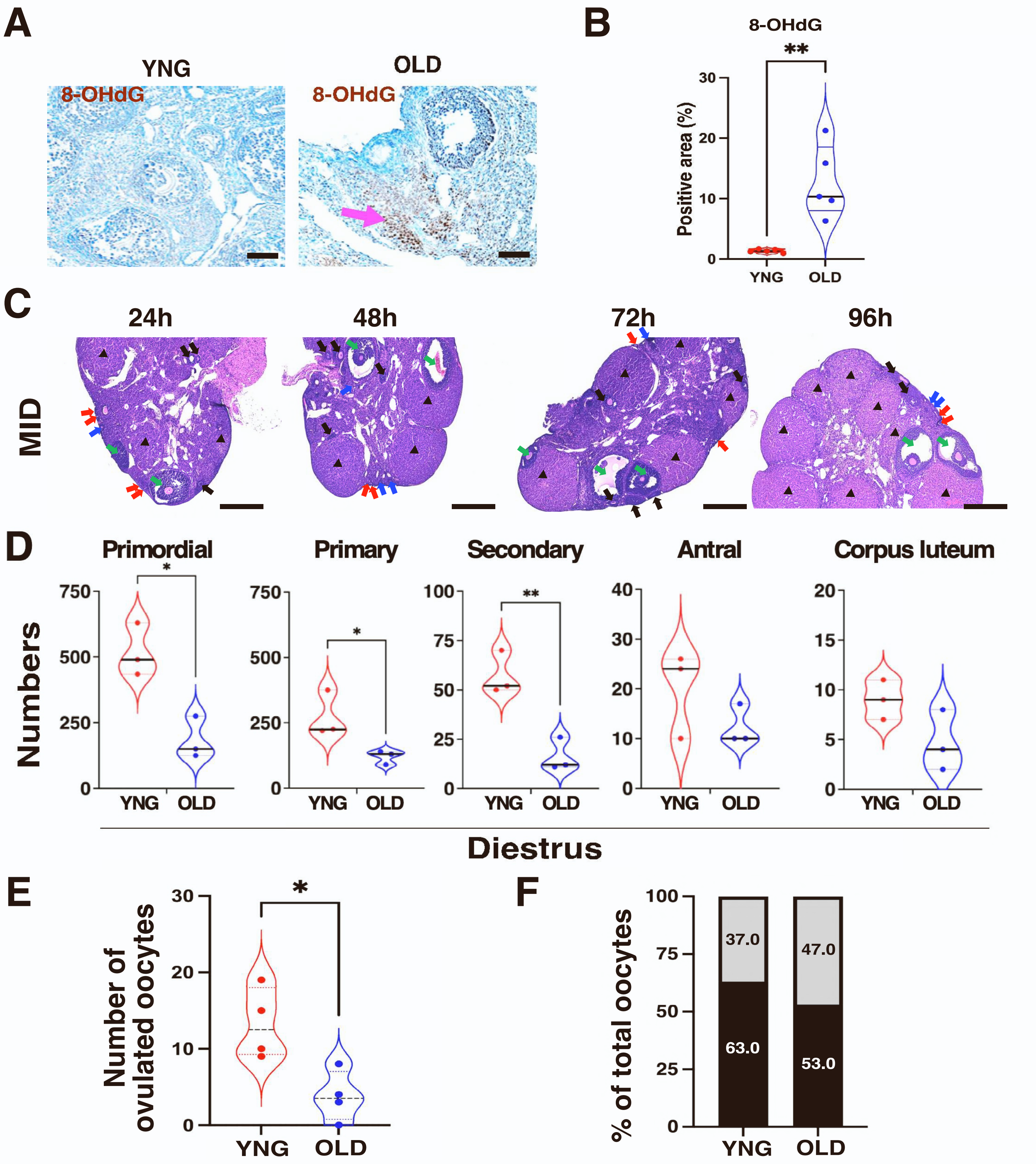

Figure S1. Follicular development and ovulation in aging mice

(A) Representative 8-OHdG staining of ovarian sections collected from YNG and OLD female mice. Arrows indicate positively stained cells. (B) Violin plots showing the 8-OHdG-positive area (%) relative to the total area of an ovarian section. n = 5, **P < 0.01; two-tailed Student's t-test. (C) Representative H&E staining of ovary sections of MID mice after PMSG/hCG injection. Follicles were indicated with arrows as follows: primordial (red), primary (blue), secondary (black), and antral (green). Corpus lutea were indicated with black arrowheads. Scale bars, 500 µm. (D) Violin plots show numbers of each follicle stage and corpora luteum in serial sections in YNG and OLD mice at diestrus stage. n = 3, *P < 0.05, **P < 0.01; two-tailed Student's t test. (E) The number of ovulated oocytes from YNG and OLD mice at 16h after PMSG/hCG injection. (F) Bar graphs show rates of mature and immature oocytes in total oocytes. YNG: n = 51, OLD: n = 15.

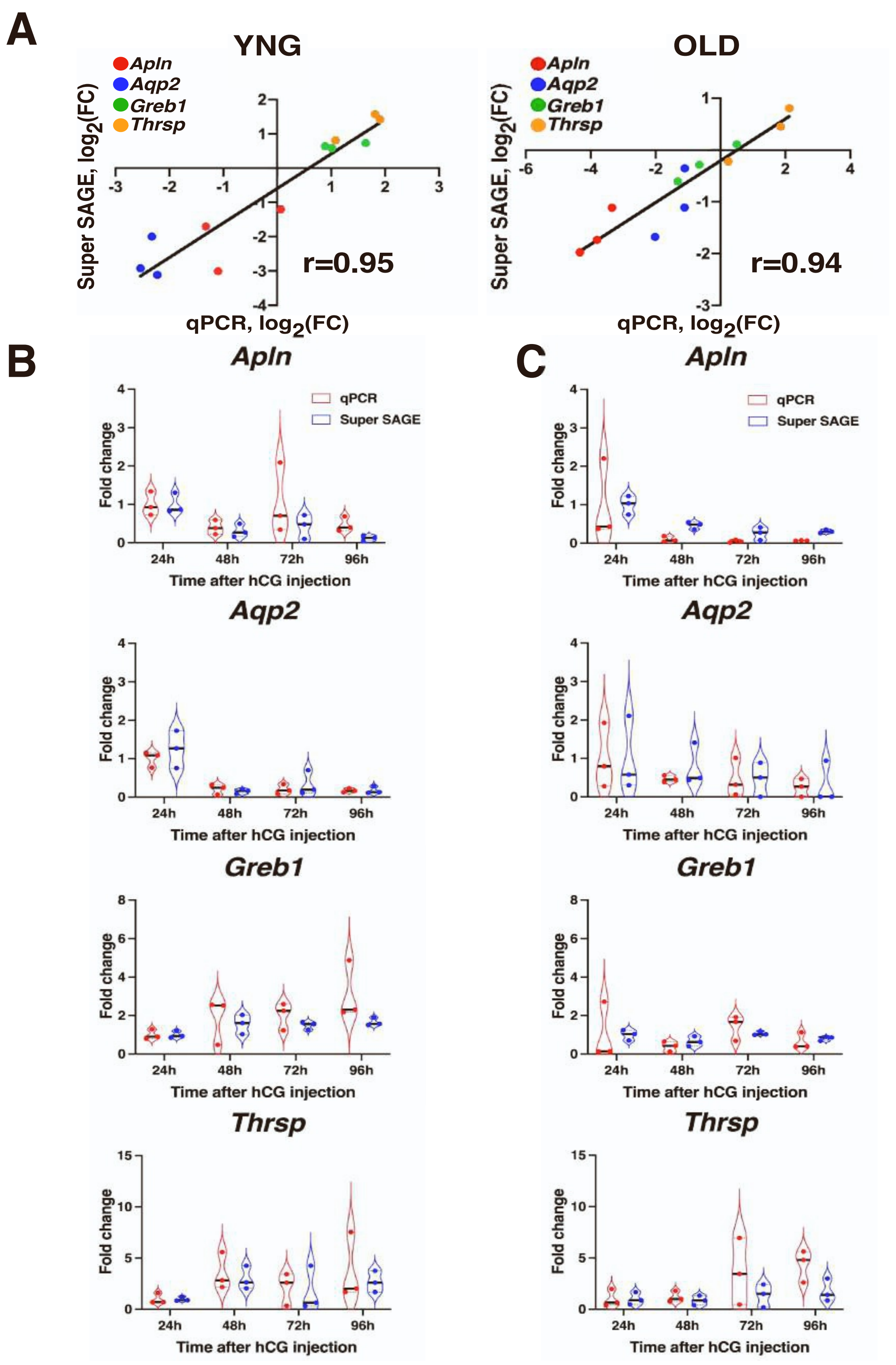

Figure S2. Correlation of mRNA expression level between whole transcriptome analysis (WTA) and qPCR
(A) mRNA expression in WTA with superSAGE (counts per million) and qPCR (relative value using *Gapdh*) were plotted to examine the correlation. Ratios (48 h/24 h, 72 h/24 h, and 96 h/24 h) of mRNA levels in the indicated genes were log2 transformed and plotted. n = 3, Correlations were valued with the Pearson correlation coefficient (r). (B and C) Violin plots showed that time course mRNA expressions the 4 genes measured using WTA with superSAGE (red) and qPCR (blue) in YNG (B) and OLD mice (C). The relative expression levels at each time point were normalized to the level at 24 h.

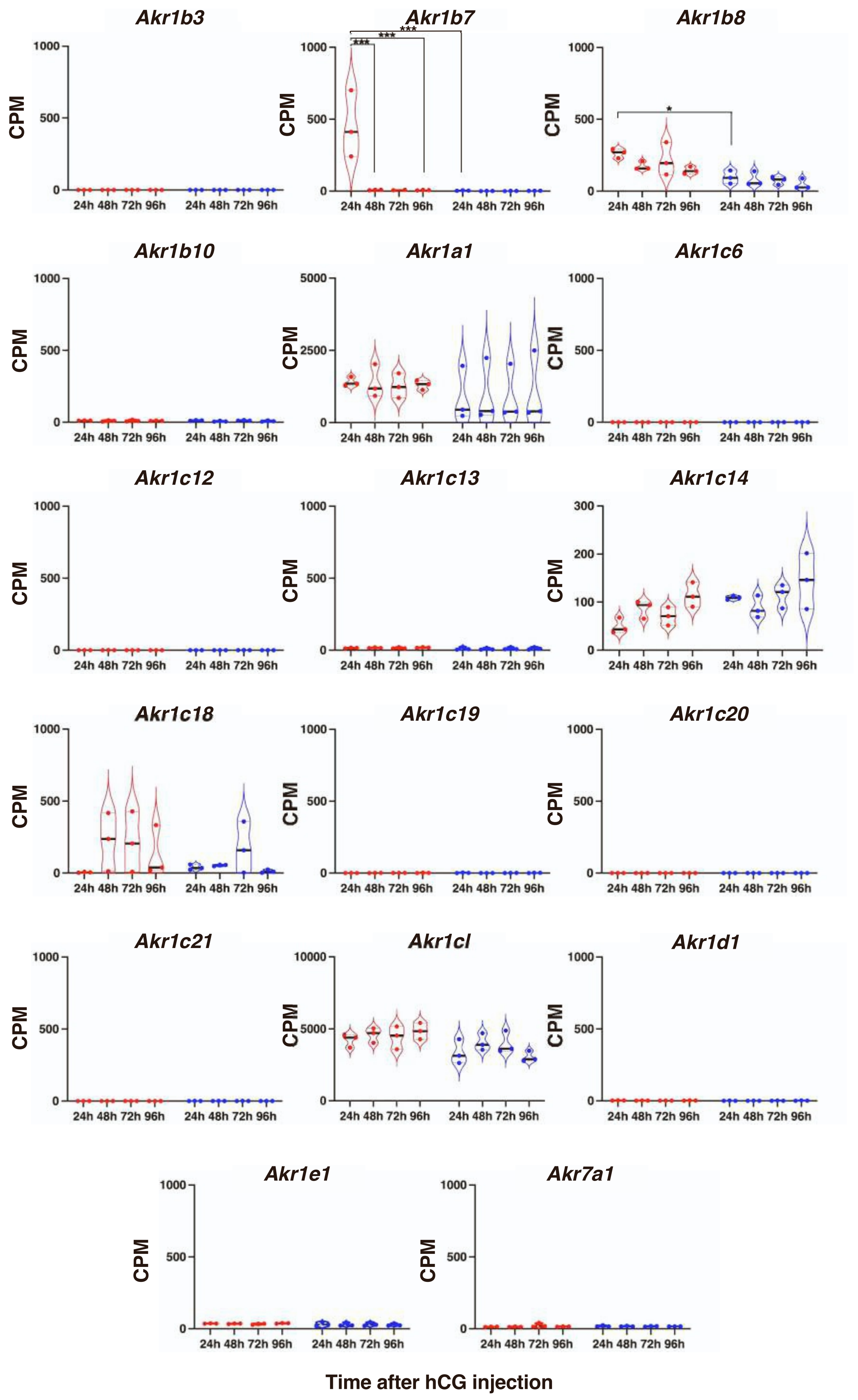

Figure S3. mRNA expression levels of aldo-keto reductase superfamily in ovarian aging
mRNA expression levels of the aldo-keto reductase superfamily in YNG and OLD ovaries were analyzed using the superSAGE method at the indicated times after PMSG/hCG injection. Expression levels are displayed as CPM. n = 3. Two-way ANOVA followed by Tukey's multiple comparison test; *P < 0.05, ***P < 0.001.

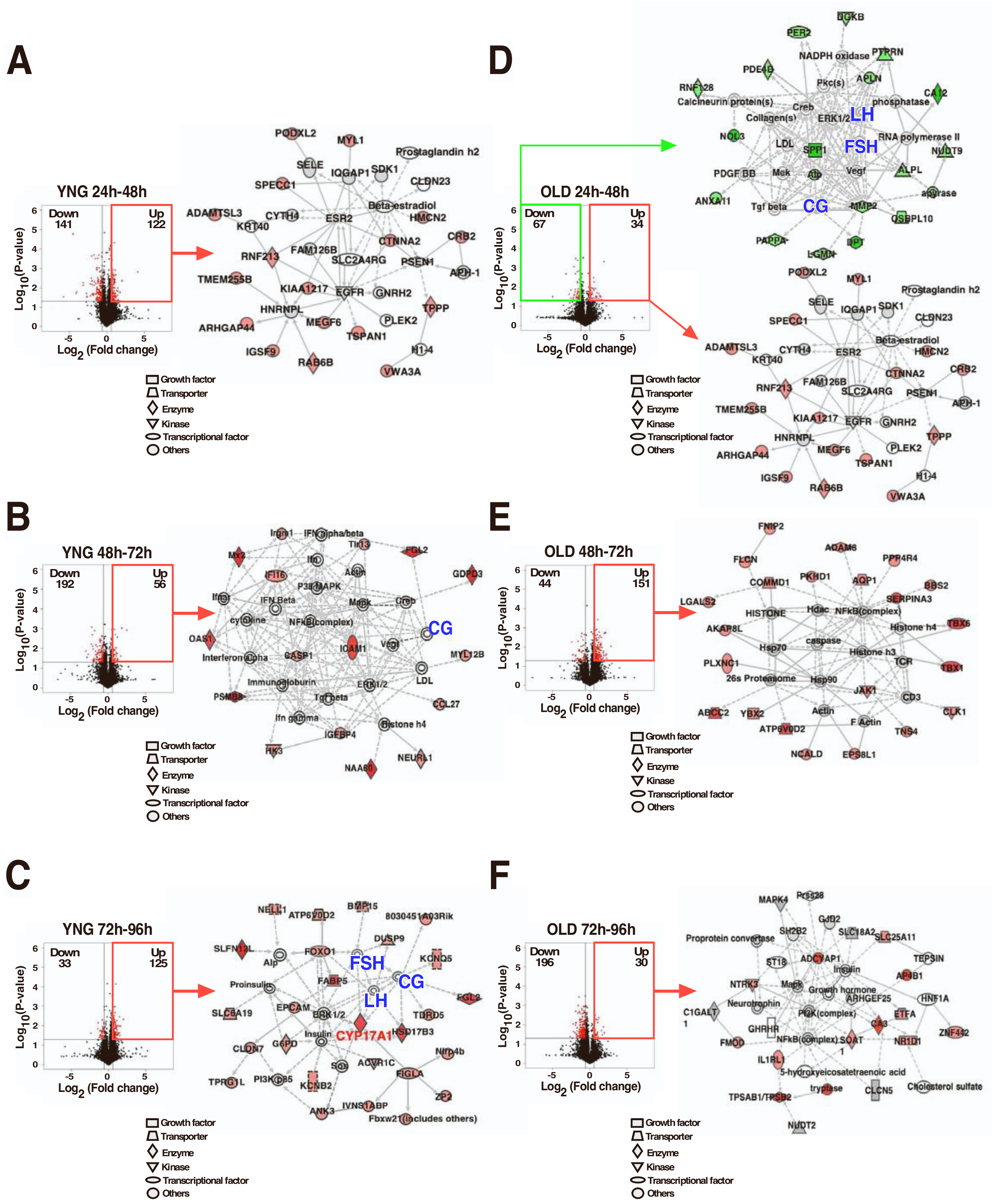

Figure S4. IPA network analysis during estrous cycle after ovulation stimulation in ovarian aging
Volcano plots indicate upregulated (red) or downregulated genes (green) with a 1.5-fold change having P value < 0.05 at the indicated times in YNG (A–C) and OLD (D–F). The regulated genes at each time point were analyzed using IPA network analysis, and each first network is shown.

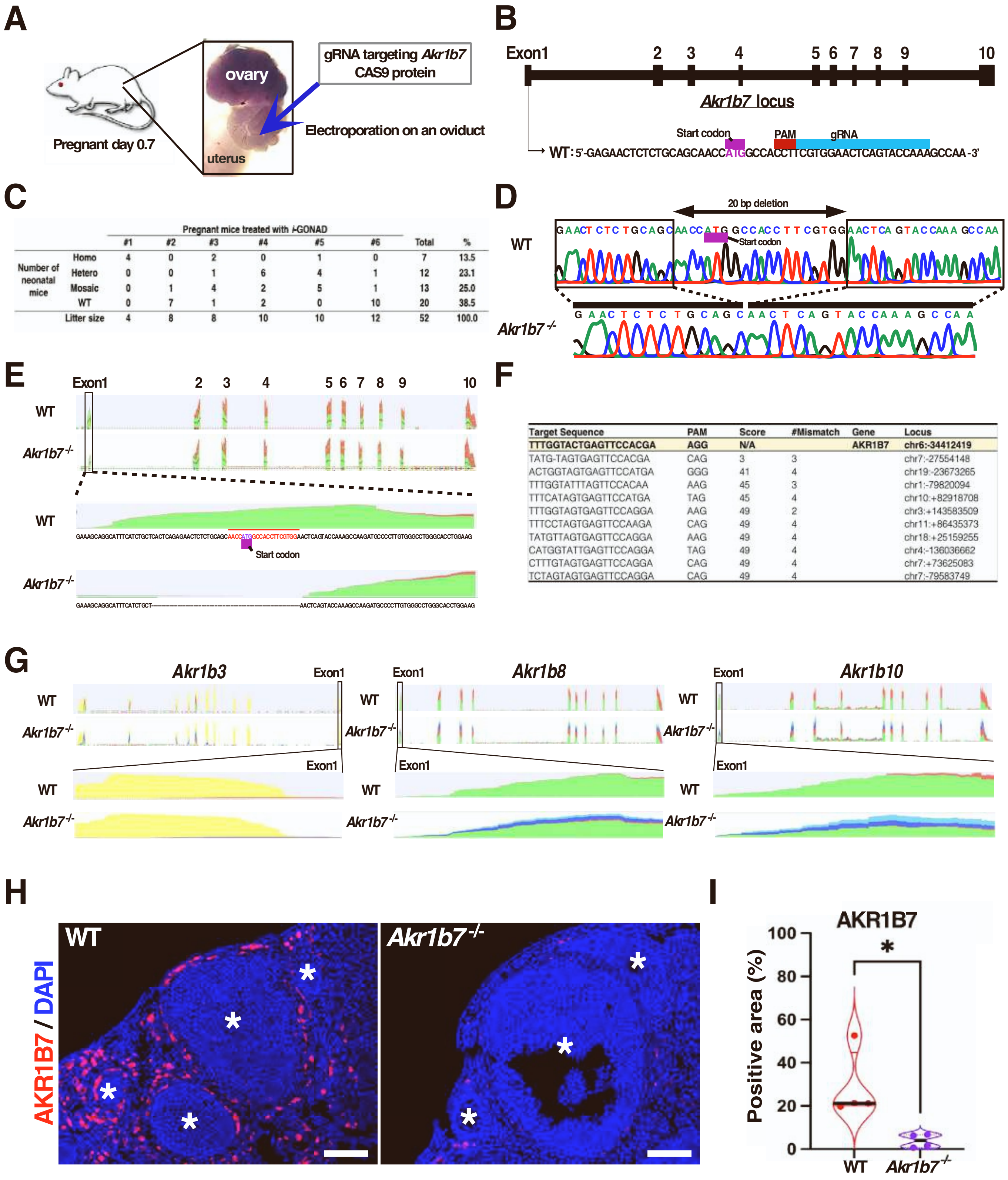

Figure S5. Generation of *Akr1b7*-deficient mice using the i-GONAD method

(A) The gRNA targeting around the start codon of *Akr1b7* and CAS9 protein was injected and electroporated on the oviduct of ICR female mice at pregnant day 0.7. (B) Genomic *Akr1b7* locus edited by the complex of *Akr1b7*-gRNA and CAS9 is shown. (C) Table shows the genotyping in pregnant mice treated with i-GONAD method. (D) Chromatograms from Sanger sequencing show DNA sequences of PCR products amplified from genomic DNA, including the *Akr1b7*-gRNA target site. Peaks on the chromatograph, green for A, red for T, blue for C, and black for G. (E) Mapping data show reads mapped on whole exon regions of *Akr1b7* with RNA-seq in WT and *Akr1b7*$^{-/-}$ (upper panel), and the data are enlarged in exon1 region of *Akr1b7* (lower panel). (F) Off-target sequences were analyzed with software, CRISPR-Cas9 gRNA checker (Integrated DNA Technologies), and the top 10 off-target regions using the guide RNA targeting of *Akr1b7* were shown. A lower score indicates a higher risk of genome editing. (G) Mapping of RNA-seq reads is shown on the whole exon (upper) and exon-1(lower) in *Akr1b3* , *Akr1b8* , and *Akr1b10* . Colored reads indicate single reads mapped in the forward direction (green), in the reverse direction (red), and paired reads (blue). Yellow reads indicate nonspecific mapping that has identical sequences to other genes. (H) Sections of immunofluorescence show staining of AKR1B7 in ovaries collected from WT and *Akr1b7*$^{-/-}$ female mice at 24 h after PMSG/hCG injection. Asterisks indicate a growing follicle belonging to the secondary or antral stage. Scale bars, 200 µm. (I) Violin plot show AKR1B7-positive area (%) in total area of an ovarian section in Figure S5H. n = 5, *$P < 0.05$; two-tailed Student's t-test.

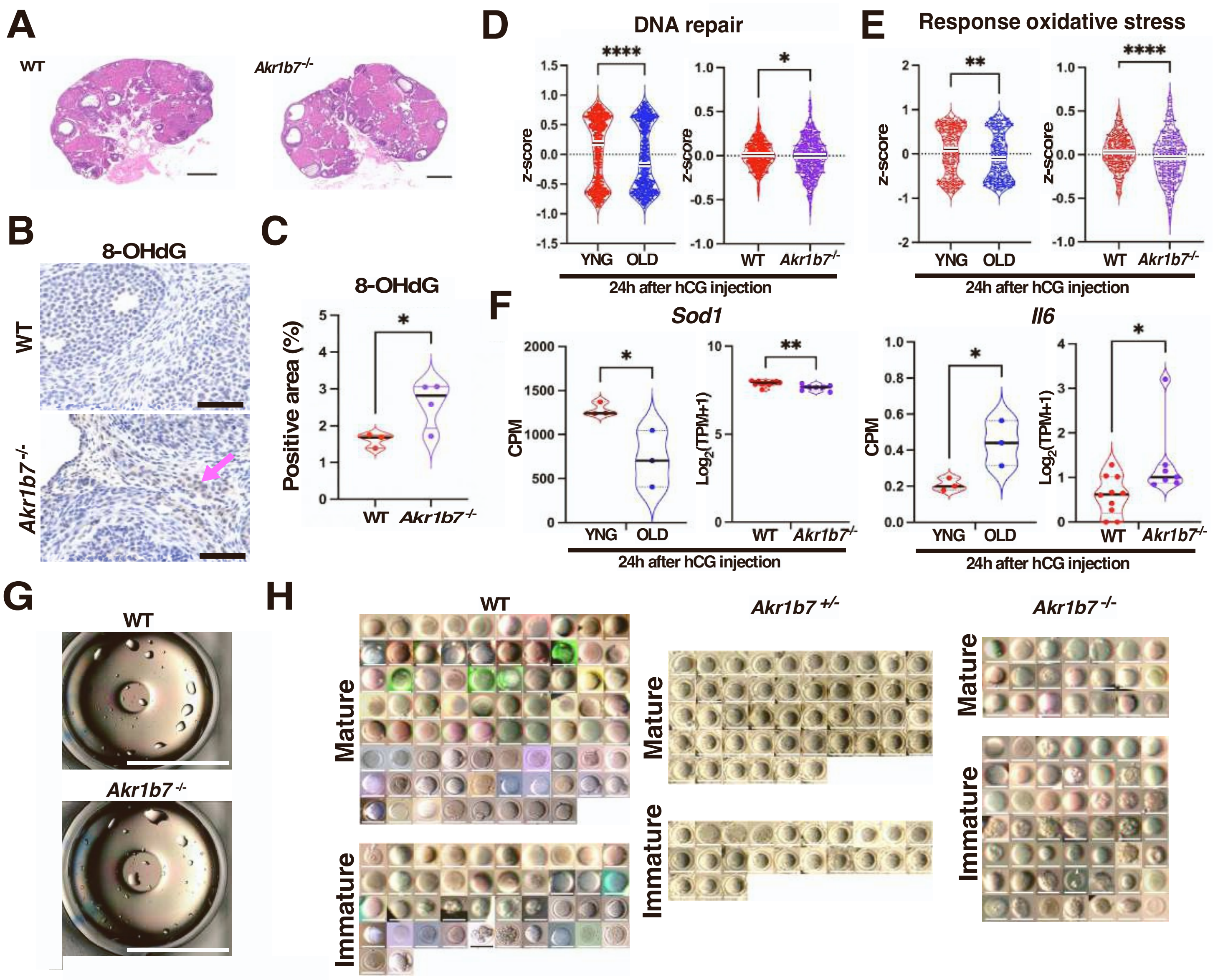

I

Genotyoe	Mouse	Immature	Mature	Total number
WT	1	16	41	57
	2	11	9	20
	3	6	7	13
	4	6	10	16
	5	3	11	14
	Total	42	78	120
Akr1b7$^{+/-}$	1	6	27	33
	2	7	9	16
	3	10	10	20
	Total	23	46	69
Akr1b7$^{-/-}$	1	16	11	27
	2	25	1	26
	3	8	9	17
	Total	49	21	70

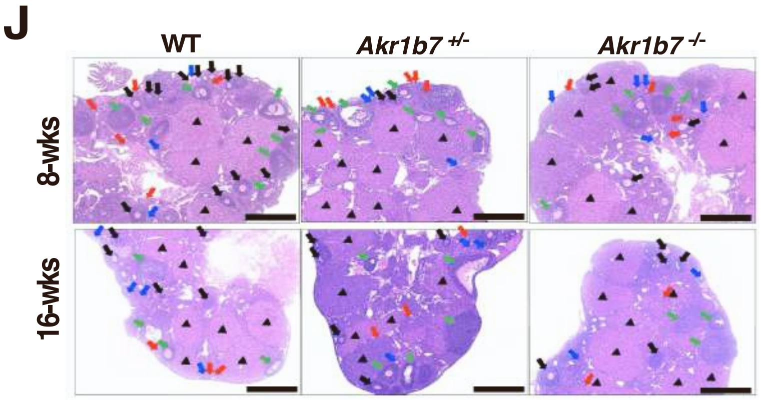

Figure S6. Expression of senescence markers and follicular development in *Akr1b7*-deficient mice after ovulation stimulation

(A) H&E-stained sections of ovaries of YNG WT and *Akr1b7*$^{-/-}$ mice. (B) Representative 8-OHdG staining of sections from ovaries collected from WT and *Akr1b7*$^{-/-}$ mice at the diestrous stage. Arrows indicate positively stained cells. Scale bars, 50 μ m. (C) Violin plots show a 8-OHdG-positive area (%) in the total area of an ovarian section. n = 3–4, *P < 0.05; two-tailed Student's t-test. (D, E) Z-score values of marker gene expressions associated with DNA repair (D) and Response to oxidative stress (E) were plotted for each group. DNA repair; 923 genes. Response to oxidative stress; 387 genes. (F) *Sod1* and *Il6* expression in YNG and OLD, WT and *Akr1b7*$^{-/-}$ ovaries at 24 h after 5 IU PMSG/hCG injection. YNG, OLD; n = 3. WT; n = 10. *Akr1b7*$^{-/-}$; n = 7. *P < 0.05, **P < 0.01; two-tailed Student's t-test. (G) Representative image of a culture plate showing wells containing oocytes observed in Figure 4G and Figure S6H. Scale bars, 10 mm. (H) The complete set of ovulated oocytes from YNG WT, *Akr1b7*$^{+/-}$, and *Akr1b7*$^{-/-}$ mice observed in Figure 4G. Scale bars, 100 μm. (I) Number of immature and mature oocytes collected from individual WT, *Akr1b7*$^{+/-}$ and *Akr1b7*$^{-/-}$ female mice observed in Figure 4G. (J) Representative H&E staining of ovary sections of 8-wks or 16-wks WT, *Akr1b7*$^{+/-}$, and *Akr1b7*$^{-/-}$ mice 24h after PMSG/hCG injection. Follicles were indicated with arrows as follows: primordial (red), primary (blue), secondary (black), and antral (green). Corpus lutea were indicated with black arrowheads. Scale bars, 500 μm.

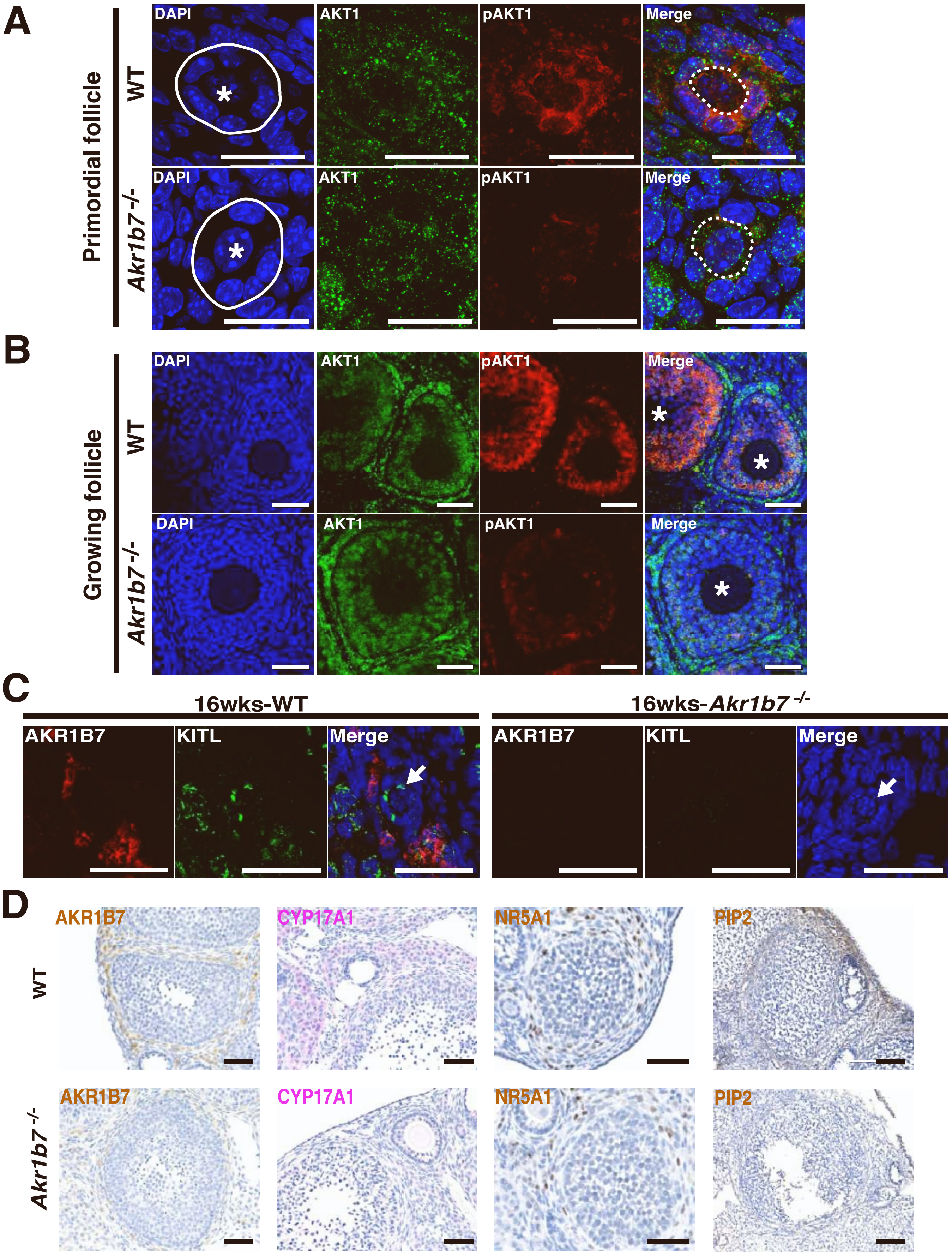

Figure S7. Confirmation of factors regulated by AKR1B7 using ovary of *Akr1b7*$^{-/-}$ mice

(A and B) Sections were prepared from ovaries of WT and *Akr1b7*$^{-/-}$ YNG mice at 24 h after 5 IU PMSG/hCG injection. (A) Circles with line and asterisks indicate primordial follicles and oocyte nuclei, respectively. Circles with dotted lines show the measured region in Figure 5K. Max intensity projections of z-stack images in 4 µm section were obtained with a 100x objective lens and 3.0x digital zoom using a confocal microscope. Nuclei-DAPI and pAKT1-Cy5 were observed by excitation at 405 and 638 nm, respectively. Scale bars, 20 µm. (B) The immunofluorescence images show staining with Nuclei-DAPI (blue), AKT1-TRITC (green), and pAKT1-Cy5 (red). Asterisks indicate a growing follicle belonging to the secondary or antral stage. n = 4, Scale bars, 50 µm. (C) Sections were prepared from 16-week-old WT and *Akr1b7*$^{-/-}$ mice ovaries at 24 h after 5 IU PMSG/hCG injection and were stained with the indicated antibodies and DAPI. Arrows show the primordial follicle. Scale bars, 20 µm. n = 4. (D) Sections were prepared from ovaries of WT and *Akr1b7*$^{-/-}$ YNG mice at 24 h after 5 IU PMSG/hCG injection and were

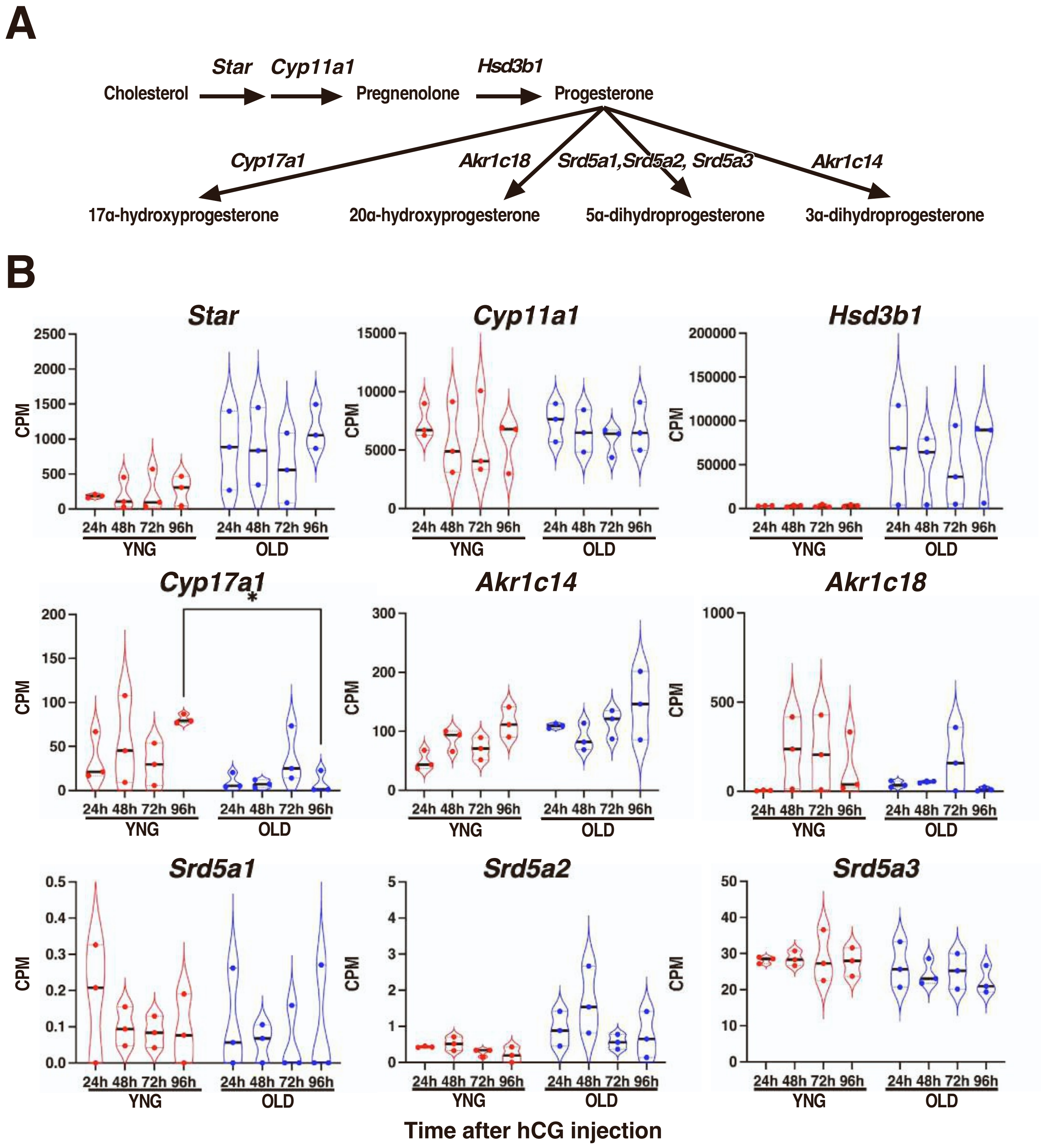

Figure S8. mRNA expressions in genes associated with progesterone metabolism during estrous cycle after ovulation stimulation

(A) Progesterone metabolism. A steroidogenic acute regulatory protein (StAR) regulates cholesterol transfer within the mitochondria. In the mitochondria, the enzyme P450 side-change cleavage (CYP11A1, also known as P450scc) converts cholesterol into pregnenolone, which is further transformed into progesterone in the endoplasmic reticulum by 3-hydroxysteroid dehydrogenase (HSD3B1). Progesterone is metabolized by P450c17 (CYP17A1), 20α-hydroxysteroid dehydrogenase (AKR1C18, also known as 20α-HSD), 3-oxo-5α-steroid 4-dehydrogenase (SRD5A1, 2, and 3), and/or 3α-hydroxysteroid dehydrogenase (AKR1C14, also known as 3α -HSD). (B) mRNA expression levels of *Star*, *Cyp11a1*, *Hsd3b1*, *Cyp17a1*, *Akr1c18*, *Akr1c14*, *Srd5a1*, *Srd5a2*, and *Srd5a3* in YNG and OLD ovaries at the indicated times after 5 IU PMSG/hCG injection. n = 3. Two-way ANOVA followed by Tukey's multiple comparison test; *$P < 0.05$.

Methods S1: Supplementary STAR Methods

Resource availability

Lead contact

Further information and requests for resources and reagents should be directed to and fulfilled by the lead contact, Yoichi Mizukami (mizukami@yamaguchi-u.ac.jp).

Materials availability

Generated mouse line in this paper will be made available on request.

Laser capture microdissection

Ovaries were embedded in paraffin block, and 12 μm sections were mounted on membrane glass slides (Leica microsystems, Wetzlar, Germany) coated with 0.1% poly-L-lysine solution in H_2O (Sigma-Aldrich, St. Louis, MO). Slides were deparaffinized with xylene, stained with H&E, and loaded onto the stage of a laser microdissection apparatus (LMD6500; Leica microsystems). The granulosa or theca cell layers were captured using an infrared capture laser under a microscope, and approximately 50 layers per sample were collected. The RNeasy formalin-fixed paraffin-embedded kit (Qiagen, Hilden, Germany) was used to extract total RNA.

Total RNA isolation and quantitative polymerase chain reaction analysis (qPCR)

qPCR was performed as previously described.[1,2] In brief, the RNeasy Mini Kit was used to isolate total RNAs from each ovary according to the manufacturer's protocol. After the reverse transcription procedure, messenger RNA (mRNA) expression values were measured using the QuantiTect SYBER Green polymerase chain reaction (PCR) kit (Qiagen) with a Rotor-Gene 6000 (Qiagen) or CFX384 (Bio-Rad, Hercules, CA). Primers used for qPCR are listed in Table S1. The relative quantity of each gene was normalized to that of *Gapdh* or *Actb* gene expression.

Whole transcriptome analysis (WTA) by serial analysis of gene expression (SAGE) method

Total RNA was extracted from the ovaries of mice by using the RNeasy Mini Kit (Qiagen). The SOLiD™ SAGE™ Kit barcoding adapter module (Thermo Fisher Scientific) was used to construct libraries of 27-bp tags adjacent to the 3′ends of mRNAs according to the manufacturer's protocol. Each library was amplified by PCR with a different barcoding primer using the SOLiD RNA Barcoding Kit and purified using the Pure-Link PCR Micro Kit (Thermo Fisher Scientific). The cDNA concentration of each library was measured by qPCR using a Rotor-Gene 6000 (Qiagen) and adjusted to 500 pM for optimal input into the emulsion PCR. Emulsion PCR for the enrichment of libraries was performed according to the protocol of SOLiD™ EZ Bead System (Thermo Fisher Scientific) as described previously.[3] SOLiD5500 (Thermo Fisher Scientific) was used to sequence

libraries of 27-bp tags. CLC genomics workbench (Qiagen) was used to trim the sequencing reads and align them to a reference mouse genome (GRCm38), as previously described.[4] Reads mapped to exon regions of each gene were counted. mRNA abundance was expressed as tag counts per million (CPM). Differentially expressed genes were defined as those with an absolute value of fold change >1.5 and normal $P < 0.05$ between a pair of samples. A volcano plot plotting the value of $\log_2$ (fold change) against $-\log_{10}$ (P value) was created using R version 3.3.0. Gene expression differences with the largest variance were visualized with a heatmap using JMP pro14 (SAS Institute Japan, Tokyo, Japan). To validate the intragroup homogeneity among age and time points, principal component analysis (PCA) was performed using JMP pro14. Network speculation and functional annotation of differentially expressed genes were performed using ingenuity pathway analysis (IPA) (Qiagen).

WTA by RNA-seq analysis

RNA-seq analysis was performed as previously described.[5-9] The RNeasy Mini Kit (Qiagen) was used to extract total RNA from the ovaries of mice, and mRNA was purified using Oligo dT beads (NEBNext Poly (A) mRNA magnet Isolation Module, New England Biolabs, NEB, Ipswitch, MA). The NEBNext Ultra II RNA Library Prep Kit (NEB) and NEBNext Multiplex Oligos for Illumina (NEB) were used to generate cDNA libraries for Illumina sequencing. An Agilent 2200 TapeStation (D1000, Agilent, Santa Clara, CA) was used to evaluate the quality and concentration of the libraries. The confirmed libraries were mixed in equal molecular amounts for clustering and sequencing on an Illumina Next-seq500 DNA sequencer with a 75 bp paired-end cycle sequencing kit (Illumina, San Diego, CA). Trimmed reads were mapped to the mouse reference genome GRCm38 release-92 using the default settings. For pathway analysis, factor loadings were calculated with the PCA of the JMP Pro version 14.0 software, the top 200 genes were selected, and the pathway analysis for the detected genes was examined using IPA (Qiagen). In the gene set analysis using z-score, gene sets obtained from a previous report[10] and GO terms (DNA repair; GO:0006281, GO:0006974, GO1990391, GO:0006260, and GO0000077, Response to oxidative stress; GO:0006979) were used.

Measurement of enzymatic activity

The cytosol extracts of ovaries in 0.1 M sodium phosphate buffer (pH 6.6) containing 1 mM dithiothreitol and protease inhibitor cocktail (Sigma-Aldrich) were collected by centrifugation at 105,000 x g for 60 min at 4ºC. The standard reaction mixture consisted of 0.1 M sodium phosphate buffer (pH 6.6), 1 mM isocaproaldehyde (Santa Cruz Biotechnology, Dallas, TX), or 640 μM 4-hydoroxynonenal (Santa Cruz Biotechnology) as a substrate, and 1 mM NADH (Sigma-Aldrich). The reaction mixture was prewarmed at 25ºC for 10 min in a 96-well plate (#Clear black plate; Greiner Bio-One, Kremsmünster, Austria), and the reaction was initiated by adding cytosolic extract.

The reductase activity for each substrate was determined by the consumption rate of NADH at 340 nm for 20 min, with 405 nm as a reference, using a FlexStation 3 microplate reader (Molecular Devices, San Jose, CA). Enzymatic characterization was performed using Lineweaver-Burk plots as described previously.[11,12] Values of the reductase activity were normalized by the total protein concentration of cytosol extracts determined using the method of Bradford (Bio-Rad).

Measurements of $PGF_{2\alpha}$

Whole ovaries were homogenized with a chilled bead mill at 3,200 rpm for 30 s in 0.1 M phosphate buffer (pH 7.4) containing 1 mM EDTA and 10 μM indomethacin. A $PGF_{2\alpha}$ enzyme immunoassay kit (Cayman Chemical, Ann Arbor, MI) was used to measure the concentrations of $PGF_{2\alpha}$ in the supernatants according to the manufacturer's instructions.[13] A FlexStation 3 Micro plate reader (Molecular devices) was used to measure the absorbance at a wavelength of 405 nm. The total protein content of the supernatant was determined using the method of Bradford (Bio-Rad).

Immunohistochemical staining

Ovaries were fixed in 4% paraformaldehyde at 4˚C overnight. The tissues were embedded in paraffin blocks and cut into 4 μm thick tissue sections. Sections were immunostained, reacted with 3,3′-diaminobenzidine (DAB) chromogen, and counterstained with hematoxylin as previously described.[4,14] Primary antibodies used for immunohistochemistry were anti-human AKR1B10 rabbit polyclonal antibody with an identical epitope sequence against mouse AKR1B7 (Thermo Fisher Scientific, #PA5-22036, 1:1500) and anti-DNA/RNA damage mouse monoclonal antibody (Abcam, Cambridge, UK, ab62623, 1:2000–1:8000). Quantitative analysis of AKR1B7 expression in sections was performed using digital images. The resulting images were divided into individual DAB and hematoxylin images by using a color deconvolution plugin in ImageJ (National Institutes of Health). The threshold parameters were set at 130 Gy for DAB staining and 200 Gy for hematoxylin staining. The trimmed DAB and hematoxylin images were transformed to pseudocolored red and blue, respectively, and overlaid using MetaMorph imaging software (Molecular Devices). The intensity of DAB staining (red) was measured in the theca layer of each follicle, and the DAB intensity per area was calculated. The theca layer was defined as a layer of elongated cells approximately 3–5 cell layers thick, immediately adjacent to the basal lamina of an ovarian follicle.[15]

Immunofluorescence staining and 3D imaging

From 4 to 5 individual ovaries for each genotype were embedded in one paraffin block and cut into 4 μm thick tissue sections. For comparative analyses, tissue sections obtained from WT and *Akr1b7*$^{-/-}$ ovaries were mounted on the same slide. An automatic procedure was performed using Ventana Benchmark Ultra (Roche Diagnostics, Basel, Switzerland). In brief, the sections were deparaffinized

and heated at 95°C for 64 min with antigen retrieval buffer, Tris-EDTA buffer (CC1; Roche Diagnostics), or citrate buffer (CC2; Roche Diagnostics). The 1st antibody buffer containing 5% serum and 0.1% Triton X was hand-applied to the drops on the slides and incubated at 37°C for 32 min. The dilution factor in the 1st antibody buffer and retrieval buffer in the antibodies were as follows: Anti-AKR1B10 rabbit polyclonal antibody (Thermo Fisher Scientific, PA5-22036, 1:2000, CC1), anti-CYP17A1 rabbit monoclonal antibody (Abcam, ab125022, 1:100, CC2), anti-NR5A1 rabbit monoclonal antibody (Cell signaling technology, Danvers, MA, 12800S, 1:50, CC2), anti-phosphorylated (Ser473) AKT1 rabbit monoclonal antibody (Abcam, ab81283, 1:50, CC2), anti-AKT1 rabbit monoclonal antibody (Cell signaling technology, #75692, 1:20, CC2), anti-AKT (pan) rabbit monoclonal antibody (Cell signaling technology, #4691, 1:600, CC2) and anti-KITL/SCF rabbit polyclonal antibody (Abcam, ab64677, 1:400, CC1). Omni-Map anti-rabbit horseradish peroxidase (HRP; Roche Diagnostics) was incubated at 37°C for 16 min and then reacted with FITC, TRITC, or Cy5 fluorophores. Sections were coverslipped using VECTASHIELD with DAPI or a Vector TrueVIEW Autofluorescence Quenching Kit with DAPI (Vector Laboratories, Burlingame, CA). Fluorescent images were captured with a z-stack of a 4 μm thick section by using a lightning mode of STELLARIS STED confocal microscope (Leica microsystems), and 3D images were generated using Imaris software (Oxford instruments, Oxford, UK). In 3D rendering using Imaris, Nuclei-DAPI, AKR1B-Cy5, and CYP17A1-FITC signals were processed in the 3D surface mode, and NR5A1-TRITC was processed with a 3D spot object. Images were corrected at both ends of the luminance histogram. For comparative analysis, the acquisition, correction, and 3D creation settings were identical for the WT and *Akr1b7*$^{-/-}$ images.

Immunofluorescence staining of PI(4,5)P2

The ovaries were frozen in Tissue-Tek O.C.T. compound (Sakura Finetek, Tokyo, Japan). Frozen sections were cut 8 μm thick and mounted on slides as previously described.[13] The sections were fixed with acetone at -30°C for 10 min and air-dried at RT for 5 min. The slides set using Ventana Benchmark Ultra. Goat F(ab) anti-mouse IgG H&L (Abcam) was incubated at 37°C for 16 min to block endogenous mouse IgG staining. The 1st antibody buffer containing anti-PI(4,5)P2 mouse monoclonal antibody (1:500, Echelon Biosciences, Salt Lake City, UT), 5% BSA, and 0.5% Triton X was hand-applied to the drop on the slides and reacted at 37°C for 32 min. Omni-Map anti-mouse HRP (Roche Diagnostics) was incubated at 37°C for 8 min and then reacted with Cy5 fluorophores. The sections were coverslipped using the Vector TrueVIEW Autofluorescence Quenching Kit with DAPI (Vector Laboratories).

Quantitative analysis on immunofluorescence staining

Digital images were acquired using an all-in-one fluorescence microscope (BZ-X800, Keyence, Osaka, Japan) and analyzed using a hybrid cell counting tool on a BZ-X800 analyzer. For comparative analyses, the acquisition settings were identical for each protein dataset. The areas of the DAPI-, TRITC-, and Cy5-positive regions were measured using fluorescence signals above the threshold value on tiling images of the whole ovary. The total area of the ovarian sections was measured using the DAPI-positive area. The oviduct and fat pad surrounding the ovary were manually excluded using the trimming tool in the software. The positive area for the targeted protein in each section was normalized to the total area of the ovarian section.

Western blotting

Whole ovaries were homogenized in RIPA lysis buffer. The supernatants were collected after centrifugation. Electrophoresis and western blotting were performed after the addition of SDS sample buffer as previously described.[16-18] The Extracts were electrophoresed in 10% (w/v) polyacrylamide gels in the presence of SDS. The gels were transferred to PVDF membranes. The membrane was blocked and incubated with anti-AKT1 rabbit monoclonal antibody (Cell Signaling Technology, cat#75692, 1:1000) or anti-phosphorylated (Ser473) AKT1 rabbit monoclonal antibody (Abcam, ab81283, 1:1000) at 4°C overnight. Anti-rabbit IgG-conjugated HRP (1:10000) was incubated for 30 min at RT. Antigens were visualized using enhanced chemiluminescence detection reagents and observed using an Amarsham Imager (Cytiva, Marlborough, MA). The densities of the detected bands were measured using ImageQuant TL software (Cytiva).

Measurements of steroid hormones in serum using ELISA

Serum was separated from whole blood by centrifugation and mixed with 4x the sample volume of diethyl ether. The extracts into organic solvent were dried at 40ºC under the nitrogen stream and dissolved with enzyme-linked immunosorbent assay (ELISA) buffer (Cayman Chemical). ELISA was performed for estradiol (E2), testosterone, and progesterone (Cayman Chemical), according to the manufacturer's protocol. A FlexStation 3 microplate reader (Molecular Devices) was used to measure absorbance at a wavelength of 405 nm.

Measurements of progesterone metabolites in ovary and serum

The homogenate of the ovary or the serum obtained from whole blood was dissolved by adding 5x the sample volume of ethyl acetate that contained one ng/µL nandrolone (Tokyo Chemical Industry, Japan), which was an internal standard. After centrifugation, the organic solvent was collected and dried at 40ºC under the nitrogen stream and then dissolved in acetonitrile/Milli-Q water (50:50). Reversed-phase high-performance liquid chromatography (HPLC) was used to measure the amount of metabolites, as previously described.[11,12,19,20] HPLC was performed using an Alliance 2695

separation module (Waters, Milford, MA) and a Cosmosil 5C18-MS-II Packed Column (4.6 x 150 mm, Nacalai Tesque) with continuous monitoring for ultraviolet absorbance at 254 nm. The mobile phase was maintained with acetonitrile: Milli-Q water (0:100) for the first 5 min, changed to acetonitrile: Milli-Q water (50:50) with a linear increase for the next 5 min, held for 10 min, and then linearly increased to 100% acetonitrile for the next 10 min. The typical elution times for nandrolone, 17α-hydroxyprogesterone, 20α-hydroxyprogesterone, and progesterone were 14.1, 16.7, 19.6, and 24.2 min, respectively. For quantitative analyses of progesterone and its metabolites, Empower 3 software (Waters) was used to measure the integrated peak areas based on the standard curves for nandrolone, 17α-hydroxyprogesterone, 20α-hydroxyprogesterone, and progesterone. The total protein content of the homogenate was determined using the method of Bradford (Bio-Rad).

1. Isayama, K., Watanabe, K., Okamoto, M., Murata, T., and Mizukami, Y. (2020). Standardization of an LNA-based TaqMan assay qPCR analysis for Aspiculuris tetraptera DNA in mouse faeces. BMC Microbiol *20*, 371. 10.1186/s12866-020-02053-6.
2. Aihara, M., Yamamoto, S., Nishioka, H., Inoue, Y., Hamano, K., Oka, M., and Mizukami, Y. (2012). Optimizing high-resolution melting analysis for the detection of mutations of GPR30/GPER-1 in breast cancer. Gene *501*, 118-126. 10.1016/j.gene.2012.04.029.
3. Pandey, K., Mizukami, Y., Watanabe, K., Sakaguti, S., and Kadokawa, H. (2017). Deep sequencing of the transcriptome in the anterior pituitary of heifers before and after ovulation. J Vet Med Sci *79*, 1003-1012. 10.1292/jvms.16-0531.
4. Watanabe, K., Yamamoto, S., Sakaguti, S., Isayama, K., Oka, M., Nagano, H., and Mizukami, Y. (2018). A novel somatic mutation of SIN3A detected in breast cancer by whole-exome sequencing enhances cell proliferation through ERalpha expression. Sci Rep *8*, 16000. 10.1038/s41598-018-34290-1.
5. Kohno, M., Kobayashi, S., Yamamoto, T., Yoshitomi, R., Kajii, T., Fujii, S., Nakamura, Y., Kato, T., Uchinoumi, H., Oda, T., et al. (2020). Enhancing calmodulin binding to cardiac ryanodine receptor completely inhibits pressure-overload induced hypertrophic signaling. Commun Biol *3*, 714. 10.1038/s42003-020-01443-w.
6. Muto, J., Fukuda, S., Watanabe, K., Dai, X., Tsuda, T., Kiyoi, T., Kameda, K., Kawakami, R., Mori, H., Shiraishi, K., et al. (2023). Highly concentrated trehalose induces prohealing senescence-like state in fibroblasts via CDKN1A/p21. Commun Biol *6*, 13. 10.1038/s42003-022-04408-3.
7. Honda, M., Shimizu, F., Sato, R., Mizukami, Y., Watanabe, K., Takeshita, Y., Maeda, T., Koga, M., and Kanda, T. (2023). Jo-1 Antibodies From Myositis Induce Complement-Dependent

Cytotoxicity and TREM-1 Upregulation in Muscle Endothelial Cells. Neurol Neuroimmunol Neuroinflamm *10*. 10.1212/NXI.0000000000200116.

8. Sato, R., Shimizu, F., Kuwahara, M., Mizukami, Y., Watanabe, K., Maeda, T., Sano, Y., Takeshita, Y., Koga, M., Kusunoki, S., and Kanda, T. (2023). Autocrine TNF-alpha Increases Penetration of Myelin-Associated Glycoprotein Antibodies Across the Blood-Nerve Barrier in Anti-MAG Neuropathy. Neurol Neuroimmunol Neuroinflamm *10*. 10.1212/NXI.0000000000200086.
9. Shimizu, F., Ogawa, R., Mizukami, Y., Watanabe, K., Hara, K., Kadono, C., Takahashi, T., Misu, T., Takeshita, Y., Sano, Y., et al. (2022). GRP78 Antibodies Are Associated With Blood-Brain Barrier Breakdown in Anti-Myelin Oligodendrocyte Glycoprotein Antibody-Associated Disorder. Neurol Neuroimmunol Neuroinflamm *9*. 10.1212/NXI.0000000000001038.
10. Yang, Q., Chen, W., Cong, L., Wang, M., Li, H., Wang, H., Luo, X., Zhu, J., Zeng, X., Zhu, Z., et al. (2024). NADase CD38 is a key determinant of ovarian aging. Nat Aging *4*, 110-128. 10.1038/s43587-023-00532-9.
11. Mizukami, Y., Sumimoto, H., Isobe, R., Minakami, S., and Takeshige, K. (1994). omega-Oxidation of lipoxin B4 by rat liver. Identification of an omega-carboxy metabolite of lipoxin B4. Eur J Biochem *224*, 959-965. 10.1111/j.1432-1033.1994.00959.x.
12. Mizukami, Y., Sumimoto, H., Isobe, R., and Minakami, S. (1993). Omega-hydroxylation of lipoxin B4 by human neutrophil microsomes: identification of omega-hydroxy metabolite of lipoxin B4 and catalysis by leukotriene B4 omega-hydroxylase (cytochrome P-450LTB omega). Biochim Biophys Acta *1168*, 87-93.
13. Isayama, K., Zhao, L., Chen, H., Yamauchi, N., Shigeyoshi, Y., Hashimoto, S., and Hattori, M.A. (2015). Removal of Rev-erbalpha inhibition contributes to the prostaglandin G/H synthase 2 expression in rat endometrial stromal cells. Am J Physiol Endocrinol Metab *308*, E650-661. 10.1152/ajpendo.00533.2014.
14. Mizukami, Y., Yoshioka, K., Morimoto, S., and Yoshida, K. (1997). A novel mechanism of JNK1 activation. Nuclear translocation and activation of JNK1 during ischemia and reperfusion. J Biol Chem *272*, 16657-16662. 10.1074/jbc.272.26.16657.
15. Magoffin, D.A. (2005). Ovarian theca cell. Int J Biochem Cell Biol *37*, 1344-1349. 10.1016/j.biocel.2005.01.016.
16. Fukui, T., Ishida, K., Mizukami, Y., Shiramoto, K., Harada, H., Yamashita, A., Yamashita, S., and Matsumoto, M. (2018). Comparison of the protective effects of direct ischemic preconditioning and remote ischemic preconditioning in a rabbit model of transient spinal cord ischemia. J Anesth *32*, 3-14. 10.1007/s00540-017-2420-5.
17. Kimura, M., Mizukami, Y., Miura, T., Fujimoto, K., Kobayashi, S., and Matsuzaki, M. (2001). Orphan G protein-coupled receptor, GPR41, induces apoptosis via a p53/Bax pathway during

ischemic hypoxia and reoxygenation. J Biol Chem *276*, 26453-26460. 10.1074/jbc.M101289200.

18. Mizukami, Y., Iwamatsu, A., Aki, T., Kimura, M., Nakamura, K., Nao, T., Okusa, T., Matsuzaki, M., Yoshida, K., and Kobayashi, S. (2004). ERK1/2 regulates intracellular ATP levels through alpha-enolase expression in cardiomyocytes exposed to ischemic hypoxia and reoxygenation. J Biol Chem *279*, 50120-50131. 10.1074/jbc.M402299200.

19. Sumimoto, H., Isobe, R., Mizukami, Y., and Minakami, S. (1993). Formation of a novel 20-hydroxylated metabolite of lipoxin A4 by human neutrophil microsomes. FEBS Lett *315*, 205-210. 10.1016/0014-5793(93)81165-v.

20. Mizukami, Y., Yamada, S., Kokudo, N., Takashima, M., and Yokoyama, T. (2001). Dietary iron reduces the anti-convulsion activity of phenytoin in electroconvulsion via inhibition of brain penetration. Brain Res *915*, 112-117. 10.1016/s0006-8993(01)02836-0.
