# Supplementary material for: Reduced Akr1b7 signaling drives ovarian aging and reproductive dysfunction
Source: iScience. 2026 Jan 19;29(2):114719. doi: 10.1016/j.isci.2026.114719 (PMC12918200; doi:10.1016/j.isci.2026.114719)
Supplement: Table S1. Sequence of qPCR-primers, TaqMan probes, and guide RNA [file mmc2.pdf]

Table S1. Sequence of qPCR-primers, -TaqMan probes, and guide RNA

Assay	Target gene	Direction	Sequence 5' to 3'	Source
qPCR	*Apln*	Forward	GGCCCCATGCCTTTCTAA	FASMAC, Kanagawa, Japan
		Reverse	AGATGTGAGGGTTCCAGAGAAGA	
	Aqp2	Forward	ATGTCTCCTTCCTTCGAGCTG	
		Reverse	CCGGCTGTTGCATTGTTG	
	Greb1	Forward	AGGCCCCACTCCTTAAACATC	
		Reverse	GGTCTTGGCAGATCACACACA	
	Thrsp	Forward	CGGAAAGCACAGGAGGTGA	
		Reverse	TCCAGGTCTCGGGTTGATG	
	Gapdh	Forward	GGTGAAGGTCGGTGTGAACG	
		Reverse	CTCGCTCCTGGAAGATGGTG	
	Akr1b3	Forward	TCCCCAAGTCTGTGACACCA	
		Reverse	TCGCTGCTCACCTCAAAGTC	
	Akr1b7	Forward	GTGACACCCTCACGCATACA	
		Reverse	ATGCACGGATCTCATCAAGCA	
	Akr1b8	Forward	GACTGCGCGTATGCCTATTG	
		Reverse	GTCCAGCTTCAGATCCGTGA	
	Akr1b10	Forward	GATCCCCAAGTCTGTGACACC	
		Reverse	CAGGCAAGCGCATCTGGTA	
LMD-qPCR	*Akr1b7*	Forward	GAGGGCCTGTGACCTGTTG	
		Reverse	GCAAGTGGACCTCAGTATTCC	
	Akr1b8	Forward	GCGGGAGGACCTCTTCATTG	
		Reverse	TCTTCTCAAAGCAGGTGGGC	
	Amh	Forward	CTCGGGCCTCATCTTAACCC	
		Reverse	GAAAGGCTTGCAGCTGATCG	
	Vim	Forward	CGAAAGCACCCTGCAGTCAT	
		Reverse	GTTCAAGGTCAAGACGTGCC	
	Actb	Forward	CTTTGCAGCTCCTTCGTTGC	
		Reverse	ATGCCGGAGCCGTTGTC	
Sanger sequencing and genotyping	*Akr1b7*	Forward	CCAGGACATAAAAATGTCACCA	
		Reverse	AGCAGCTGACAACAGGTGATAA	
Affinity Plus® TaqMan probes for genotyping	*Akr1b7*- WT allele	–	/FAM/CATGGCC+ACCT+TCGTGGAAC/IBFQ/	Integrated Dna Technologies, Coralville, IA
	Akr1b7- KO allele	–	/HEX/TGCAGC+AACTC+AGTACCAAA/IBFQ/	
Guide RNA for CAS9	*Akr1b7*	–	UUUGGUACUGAGUUCCACGA	
